# *Mycobacterium tuberculosis* subverts negative regulatory pathways in human macrophages to drive immunopathology

**DOI:** 10.1371/journal.ppat.1006367

**Published:** 2017-06-01

**Authors:** Patience T. Brace, Liku B. Tezera, Magdalena K. Bielecka, Toby Mellows, Diana Garay, Shuye Tian, Lucinda Rand, Justin Green, Sanjay Jogai, Andrew J. Steele, Timothy M. Millar, Tilman Sanchez-Elsner, Jon S. Friedland, Christopher G. Proud, Paul T. Elkington

**Affiliations:** 1 NIHR Biomedical Research Centre, Clinical and Experimental Sciences Academic Unit, Faculty of Medicine, University of Southampton, Southampton, United Kingdom; 2 South Australian Health and Medical Research Institute, Adelaide, and School of Biological Sciences, University of Adelaide, Adelaide, Australia; 3 Department of Infectious Diseases and Immunity, Imperial College London, London, United Kingdom; 4 Cancer Sciences, Faculty of Medicine, University of Southampton, Southampton, United Kingdom; 5 Institute for Life Sciences, University of Southampton, Southampton, United Kingdom; Portland VA Medical Center, Oregon Health and Science University, UNITED STATES

## Abstract

Tuberculosis remains a global pandemic and drives lung matrix destruction to transmit. Whilst pathways driving inflammatory responses in macrophages have been relatively well described, negative regulatory pathways are less well defined. We hypothesised that *Mycobacterium tuberculosis* (Mtb) specifically targets negative regulatory pathways to augment immunopathology. Inhibition of signalling through the PI3K/AKT/mTORC1 pathway increased matrix metalloproteinase-1 (MMP-1) gene expression and secretion, a collagenase central to TB pathogenesis, and multiple pro-inflammatory cytokines. In patients with confirmed pulmonary TB, PI3Kδ expression was absent within granulomas. Furthermore, Mtb infection suppressed PI3Kδ gene expression in macrophages. Interestingly, inhibition of the MNK pathway, downstream of pro-inflammatory p38 and ERK MAPKs, also increased MMP-1 secretion, whilst suppressing secretion of TH_1_ cytokines. Cross-talk between the PI3K and MNK pathways was demonstrated at the level of eIF4E phosphorylation. Mtb globally suppressed the MMP-inhibitory pathways in macrophages, reducing levels of mRNAs encoding PI3Kδ, mTORC-1 and MNK-1 via upregulation of miRNAs. Therefore, Mtb disrupts negative regulatory pathways at multiple levels in macrophages to drive a tissue-destructive phenotype that facilitates transmission.

## Author summary

The mechanisms whereby *Mycobacterium tuberculosis* (Mtb) evades host immunity are well described, but Mtb must also engage the host immune response to drive tissue destruction, cavitation and transmission as part of its life cycle. We identify negative regulatory pathways that suppress pathogenic matrix metalloproteinase-1 expression in primary human macrophages, including a previously unidentified role of the MAP kinase-interacting kinase (MNK) pathway in inhibiting protease secretion. Furthermore, these pathways are suppressed in granulomas of patients with culture-proven pulmonary tuberculosis and in infected macrophages *in vitro*. Stability of mRNA encoding negative regulatory molecules is reduced, and Mtb upregulates multiple microRNAs predicted to target their 3’UTR. Together, these findings demonstrate that Mtb skews the macrophage phenotype towards tissue destruction by disrupting negative regulatory pathways.

## Introduction

Tuberculosis (TB) is a global pandemic, killing more than any other infectious disease [1], and ongoing transmission in high incidence settings impedes control measures [2]. *Mycobacterium tuberculosis* (Mtb), the causative organism, must cause lung destruction to create highly infectious individuals with pulmonary cavities who drive the pandemic [3]. Cavitation results from tissue-destructive matrix metalloproteinases (MMPs) [4], in particular MMP-1 from macrophages [5–7]. The signalling pathways driving MMP-1 expression have been described [5, 8], but relatively little is known about regulatory pathways that limit immunopathology in tuberculosis [9].

Pro-inflammatory signalling in LPS-stimulated dendritic cells is negatively regulated by the phosphoinositol-3 kinase (PI3K) signalling pathway, which inhibits IL-12 secretion and TLR signalling [10, 11]. Intracellular signalling is highly complex, with cross-talk between cascades such as the mitogen-activated protein kinase (MAPK), PI3K and MAP kinase-interacting kinase (MNK) pathways [12]. MNKs (MNK1/2) are protein kinases which phosphorylate the translation initiation factor eIF4E and are therefore thought to regulate mRNA translation [13], but have not previously been studied in TB. The precise role of MNK-mediated eIF4E phosphorylation is unclear, but is considered to differentially affect the translation of multiple mRNAs [12–14]. Expression of these intracellular signalling molecules can be regulated by microRNAs [15], and Mtb infection of macrophages can modulate this microRNA profile [16–18].

We hypothesised that negative regulatory pathways in macrophages limit excessive immunopathology in TB, and that the pathogen specifically targets them to exacerbate tissue destruction and consequently transmission. In the present study, we have identified for the first time regulatory pathways which limit MMP-1 production in human macrophages, including PI3K, AKT and mTORC1, and show that PI3K expression is reduced in pulmonary granulomas of patients with TB. Intriguingly, MNK inhibition also increases MMP-1 secretion by as yet an undescribed signalling pathway. Furthermore, Mtb infection suppresses mRNA levels of multiple regulatory pathways in macrophages via augmenting expression of several key micro-RNAs (miRNA). Therefore, the pathogen skews the macrophage response to promote tissue destruction.

## Results

### The PI3K pathway negatively regulates MMP-1 expression in primary human macrophages

**Fig 1:**
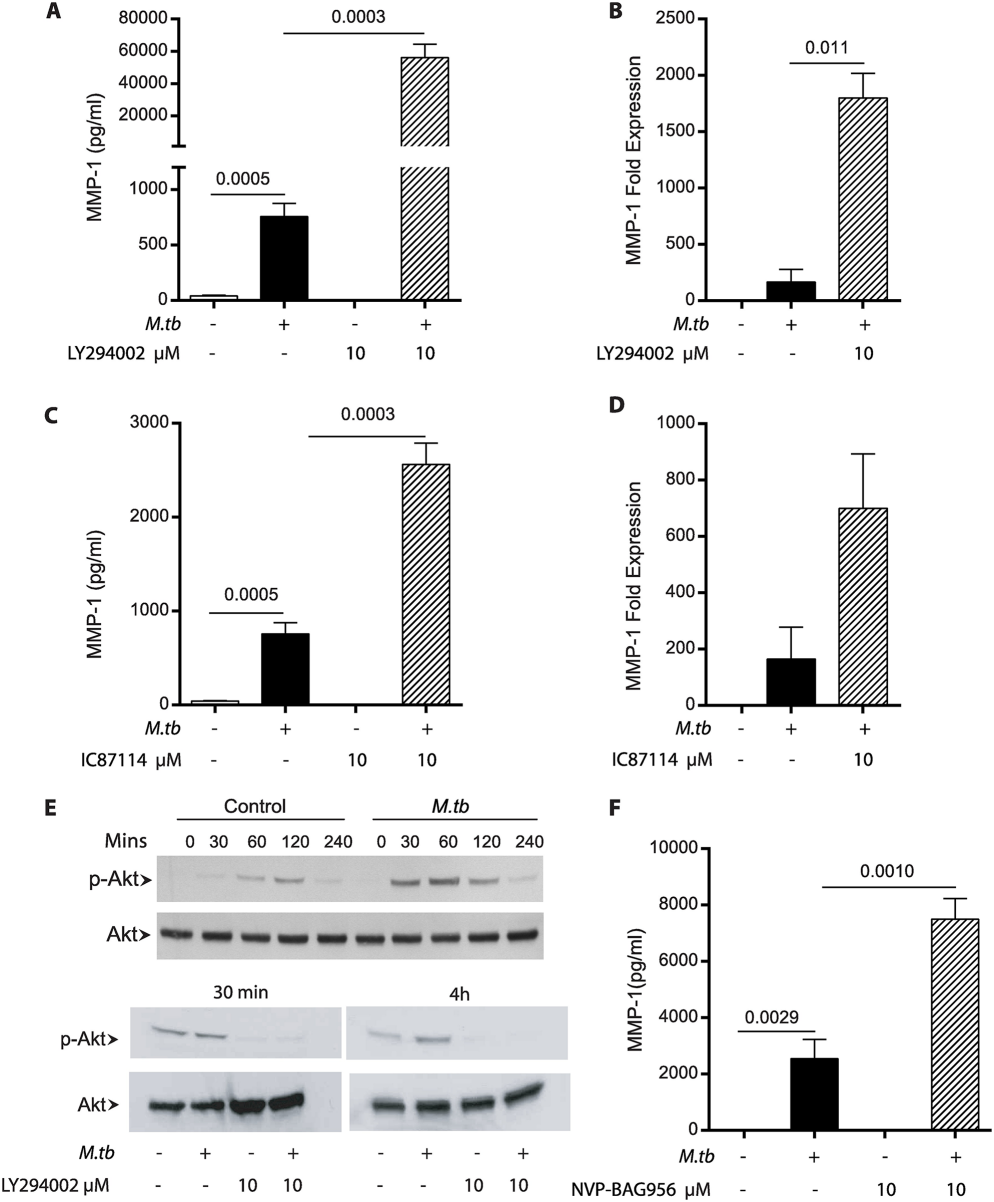
PI3K inhibition augments Mtb-driven macrophage MMP-1 secretion. **A.** Global inhibition of the PI3K kinase pathway with LY294002 significantly increases macrophage MMP-1 secretion at 72h. **B.** PI3K inhibition increases macrophage MMP-1 gene expression at 24 h. **C.** Targeted inhibition of PI3Kδ with IC87114 similarly increases MMP-1 secretion at 72h. **D.** Increased secretion with IC87114 is secondary to increased mRNA accumulation at 24h. **E.** Mtb infection increases AKT phosphorylation from 30 minutes, peaking at 60 minutes and declining after 4 h. AKT phosphorylation is completely inhibited by LY294002 at 30 minutes and 4 h. **F.** Targeted inhibition of PI3K / PDK1 with NVP-BAG956 also increases MMP-1 secretion at 72h. Data show mean and standard deviation of experiments performed in triplicate and are representative of experiments performed on a minimum of three occasions. P values are Student’s t-test.

As previously demonstrated, Mtb infection of primary human macrophages significantly increased MMP-1 secretion and expression (Fig 1A and 1B). However, global inhibition of PI3K signalling with the pan-PI3K inhibitor LY294002 further augmented Mtb-induced MMP-1 secretion (Fig 1A) and gene expression (Fig 1B). The PI3K pathway has multiple subunits, and we studied the PI3Kδ subunit which is specifically expressed in cells derived from the blood by specific inhibition with IC87114 (PI3Kδ, PI3Kγ and PI3Kβ = IC_50_ 0.5, 29 and 75μM, respectively). Consistent with the global inhibition, PI3Kδ inhibition significantly upregulated MMP-1 secretion (Fig 1C) and MMP-1 gene expression (Fig 1D). LY294002 did not suppress expression of *PIK3CD*, the gene encoding PI3Kδ, in macrophages after 24h of incubation (S1 Fig). We subsequently evaluated AKT phosphorylation, immediately downstream of PI3K within the signalling cascade. Mtb infection induced AKT phosphorylation at Ser 473 within 30 minutes, peaking at 60 minutes and was still evident at 120mins. This downstream signalling was completely inhibited by LY294002 (Fig 1E and S2 Fig). To address the question of potential off-target effects of the inhibitors, we evaluated an additional PI3K/PDK1 inhibitor, NVP-BAG956, and again demonstrated increased MMP-1 secretion after inhibition (Fig 1F). To investigate whether MMP-1 upregulation was augmented by intercellular networks, we stimulated macrophages with conditioned media from Mtb-infected monocytes. MMP-1 secretion was increased after stimulation with media from infected macrophages, demonstrating that intercellular networks can augment MMP-1 driven by Mtb infection (S3 Fig).

### PI3K inhibition globally modulates the inflammatory secretome of macrophages

**Fig 2:**
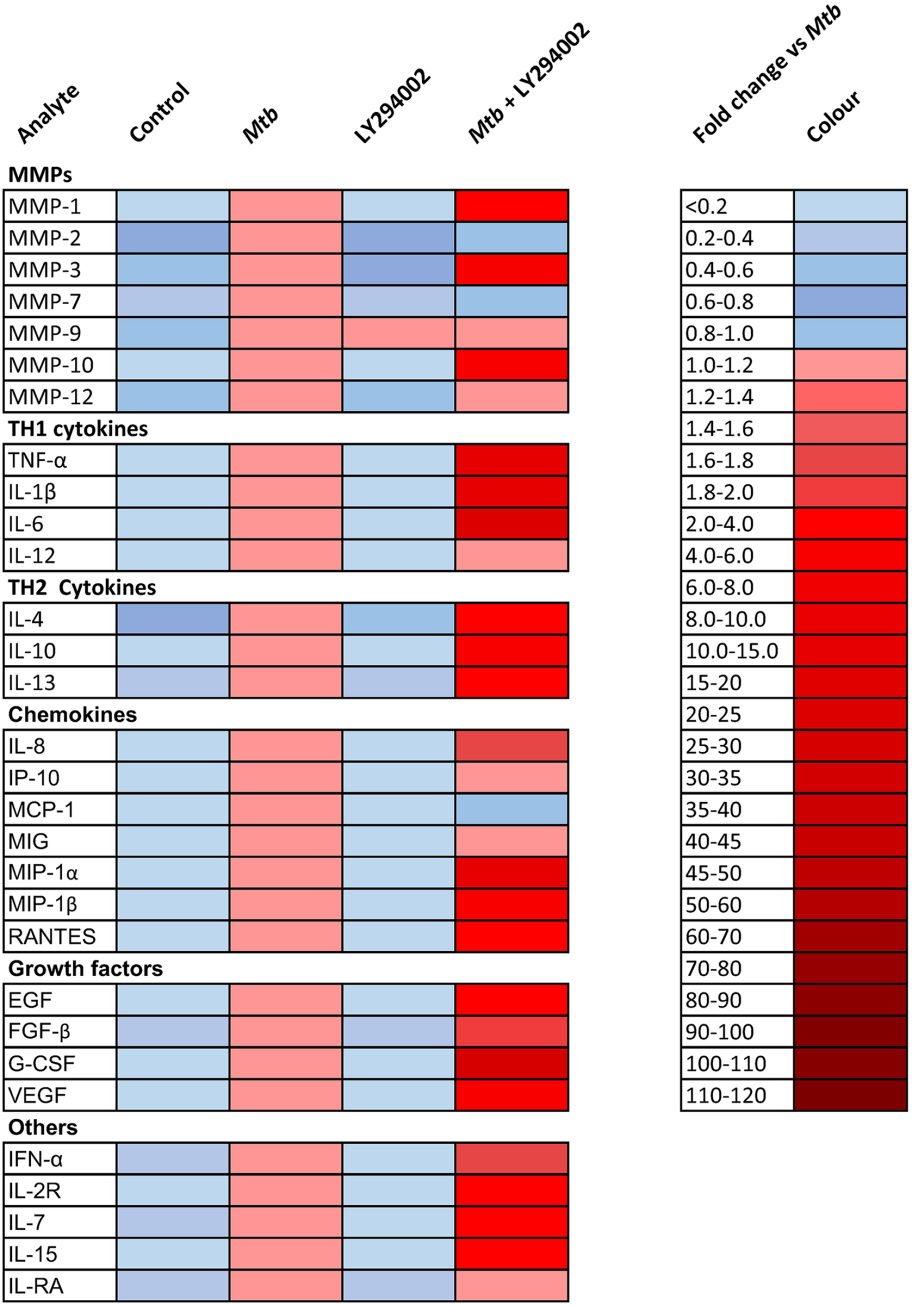
PI3K inhibition has global effects on MMP, cytokine and chemokine secretion by macrophages. Luminex profiling of MMPs, cytokines, chemokines and growth factors demonstrates that Mtb infection significantly upregulates a wide range of mediators from macrophages at 72h. Secretion of the majority of mediators was further augmented when PI3K signalling was globally inhibited. Median values normalised to Mtb-infected samples of an experiment performed in triplicate are shown, and are representative of an experiment performed on two separate occasions.

To determine whether the PI3K regulation of MMP-1 was specific or part of a more widespread phenomenon, we profiled secretion of MMPs, cytokines, chemokines and growth factors by uninfected and infected macrophages using a Luminex array. Mtb infection upregulated numerous mediators, and inhibition of PI3K signalling had an additive effect, causing further upregulation of secretion of multiple MMPs, TH_1_ and TH_2_ cytokines, chemokines and growth factors (Fig 2). In the context of PI3K pathway inhibition, the majority of pro-inflammatory mediators were upregulated.

### PI3Kδ expression is suppressed in TB lesions and in infected macrophages

**Fig 3:**
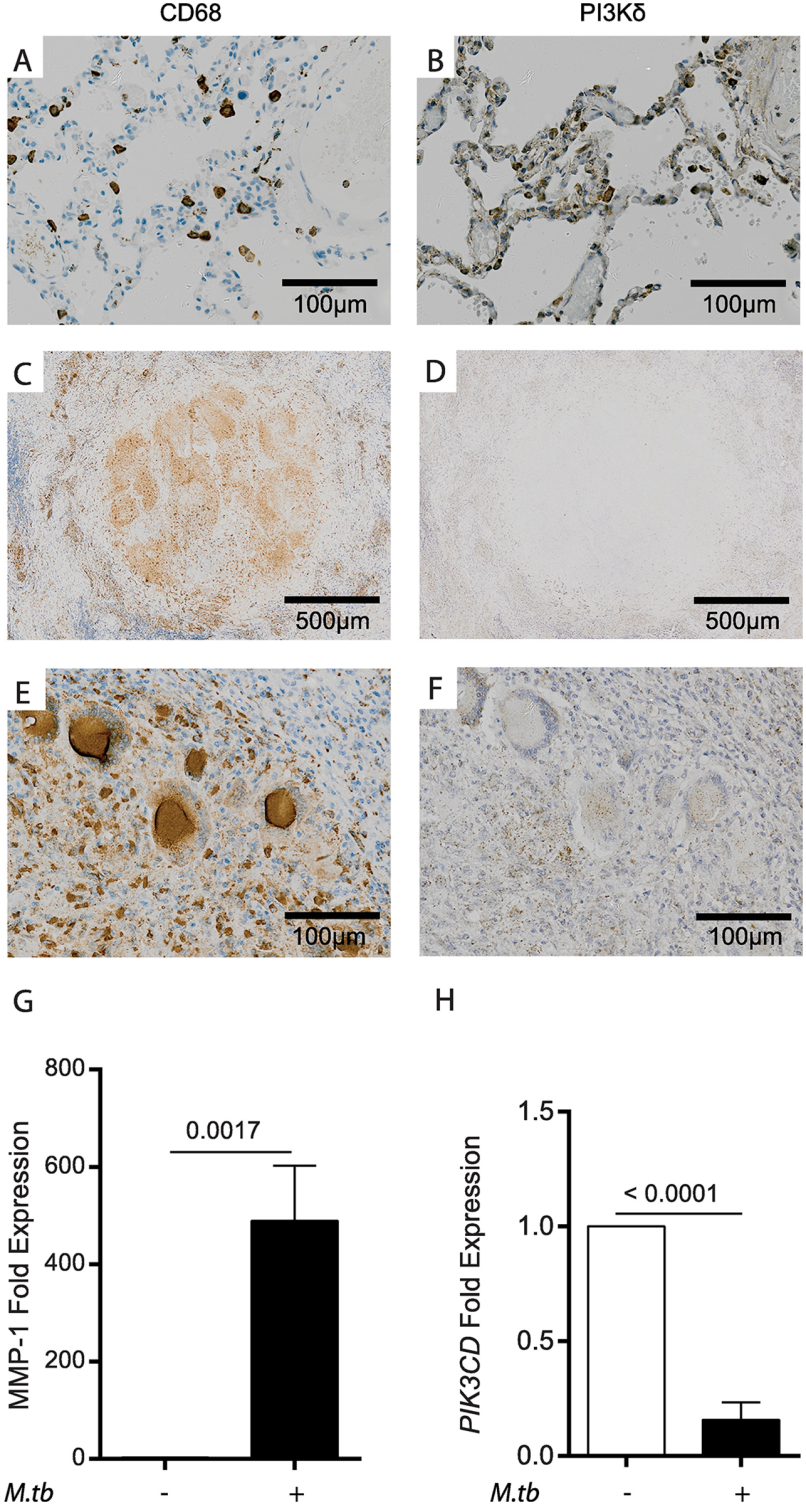
PI3Kδ expression is suppressed in human TB granulomas and infected macrophages. **A, B.** Immunohistochemistry analysis of normal human lung tissue demonstrates that alveolar macrophages express both CD68 and PI3Kδ. **C, D**. Within human lung TB granulomas, CD68 is widely expressed, but no PI3Kδ immunoreactivity is observed. **E, F.** Both epithelioid macrophages and multinucleate giant cells around TB granulomas express CD68, but neither express PI3Kδ. **G.** In primary macrophages, Mtb infection significantly upregulates MMP-1 expression at 24h, whilst (**H**) concurrently suppresses *PIK3CD* mRNA accumulation. Immunohistochemistry images are representative of six donors with histopathologically confirmed TB who had lung biopsies as part of their routine clinical care. Scale bars: A, B, E, F: 100μm, C, D: 500 μm. Cellular experiments were performed in triplicate and are representative of at least three donors. P values are t-test comparisons.

Next, we investigated PI3Kδ signalling in lung lesions of patients with confirmed pulmonary TB. Initially, we performed immunohistochemistry for phosphorylated PI3K to determine activation *in vivo*, but were unable to demonstrate immunoreactivity. Therefore, we analyzed total PI3Kδ, but again were unable to detect expression. In normal lung tissue, alveolar macrophages expressed both CD68 and PI3Kδ (Fig 3A and 3B). Within tuberculosis granulomas, CD68 expression is widespread (Fig 3C) and multinucleate giant cells also express CD68 (Fig 3E). In contrast, expression of PI3Kδ is globally absent throughout the granuloma (Fig 3D) and also absent in macrophages and multinucleate giant cells (Fig 3F). Positive controls excluded a technical cause for the absent PI3Kδ staining, and analysis of further granulomas confirmed the absence of PI3Kδ despite widespread CD68 expression (S4 Fig).

Therefore, we examined the effect of Mtb infection on macrophage PI3Kδ expression. Mtb upregulated expression of MMP-1 gene expression at 24h (Fig 3G), but in the same cells significantly suppressed expression of *PIK3CD*, the gene endcoding PI3Kδ (Fig 3H). Therefore, the increase in MMP-1 expression is accompanied by suppression of PI3Kδ in both patients and primary human macrophages. Stimulation of macrophages with conditioned media from Mtb-infected cells did not suppress *PIK3CD*, suggesting that intercellular networks were not the primary driver of *PIK3CD* suppression.

### AKT and mTORC1 negatively regulate MMP-1 expression in macrophages

**Fig 4:**
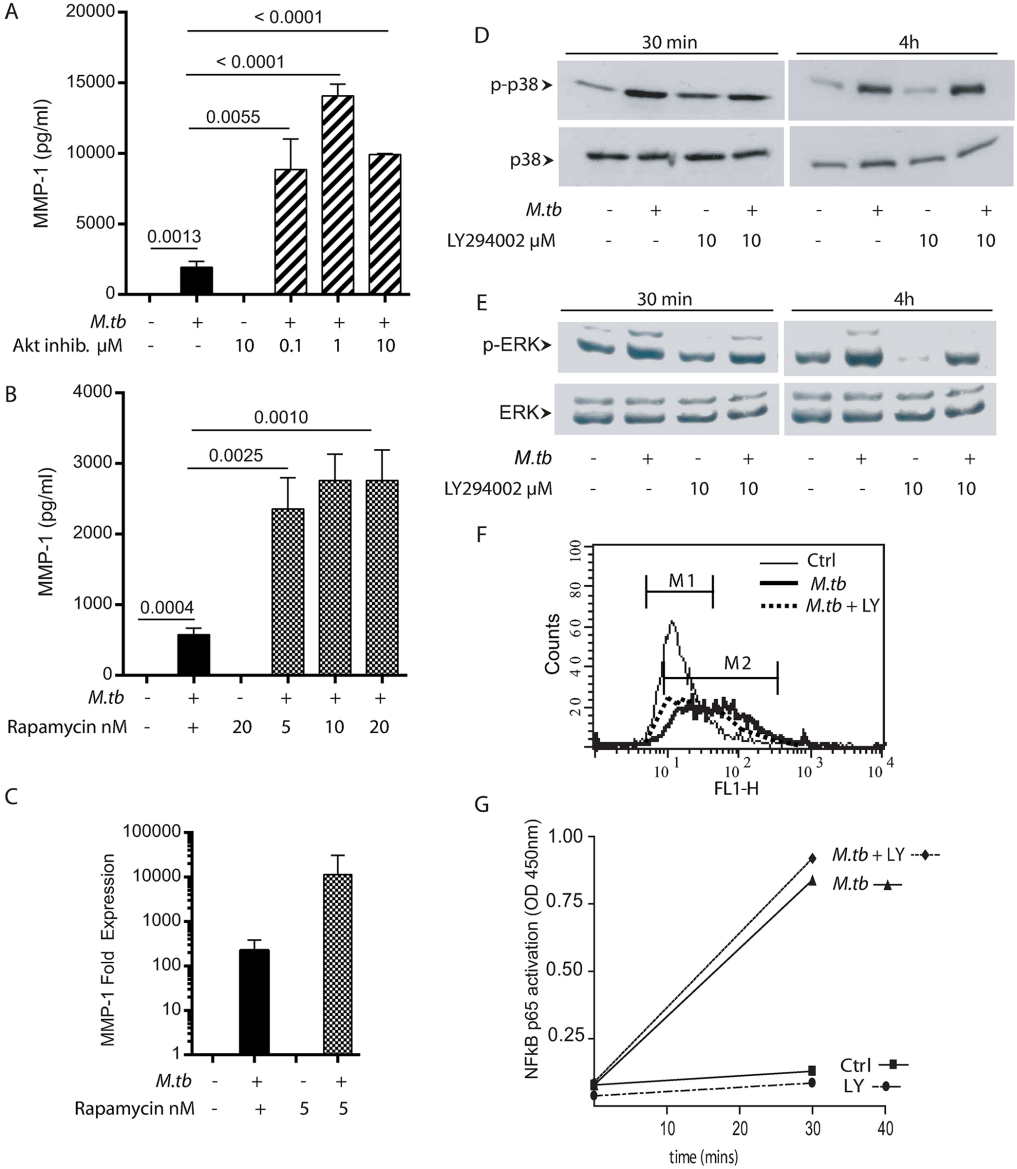
Negative regulation of MMP-1 is via the AKT and mTORC1 pathways, but the PI3K pathway does not cross talk with p38, COX-II or NFκB signalling. **A.** Inhibition of AKT significantly increases MMP-1 secretion from macrophages at 72h. **B.** Inhibition of mTORC1 signalling with rapamycin similarly augments macrophage MMP-1 secretion at 72h. **C.** MMP-1 mRNA accumulation is increased in rapamycin-treated cells at 24h. **D.** Mtb increases p38 phosphorylation in macrophages analysed by western blotting, but PI3K pathway inhibition does not affect this at 30 minutes or 4h. **E.** PI3K inhibition does not augment constitutive ERK phosphorylation. **F.** PI3K inhibition has no effect on accumulation of COX-II protein in infected macrophages at 24h, analysed by flow cytometry. **G.** Mtb increases NFκB activation, but no difference is observed after PI3K inhibition with LY294002. Experiments have been performed on three occasions (A, B, D, E) and two occasions (C, F, G). Mean and standard deviations are shown and p values are by Student’s t-test.

We then studied the signalling pathway directly downstream of PI3Kδ, which includes AKT and mTORC1. Inhibition of AKT signalling, which is phosphorylated following PI3Kδ activation, similarly upregulated MMP-1 secretion from macrophages (Fig 4A). Similarly, mTORC1 inhibition with rapamycin augmented Mtb-driven MMP-1 secretion at 72h (Fig 4B), and this associated with increased MMP-1 gene expression at 24h. Luminex profiling of MMPs and pro-inflammatory cytokines demonstrated a global effect of upregulation of MMPs and cytokines after mTORC1 inhibition (S5 Fig), as was observed for PI3K inhibition (Fig 2).

We then investigated whether these inhibitory pathways increased MMP-1 via crosstalk with the pro-inflammatory MAPK pathways, which regulate MMP-1 secretion in macrophages [8]. PI3K inhibition did not increase p38 MAPK phosphorylation at either 30 or 240 minutes (Fig 4D and S2 Fig), nor ERK MAPK phosphorylation at these time points (Fig 4E). We measured cyclo-oxygenase II (COX-II) accumulation, which regulates MMP-1 downstream of p38, but PI3K inhibition did not alter COX-II levels within infected macrophages (Fig 4F). Finally, we investigated whether PI3K linked with NFκB signalling. Mtb increased the nuclear translocation of p65 in macrophages, but this was not altered by LY294002, suggesting that crosstalk was not occurring at this level (Fig 4G). Therefore, the PI3K pathway suppresses MMP-1 expression independent of regulating the MAPK, COX-II or NFκB axes.

### The MNK pathway also negatively regulates MMP-1 in infected macrophages

**Fig 5:**
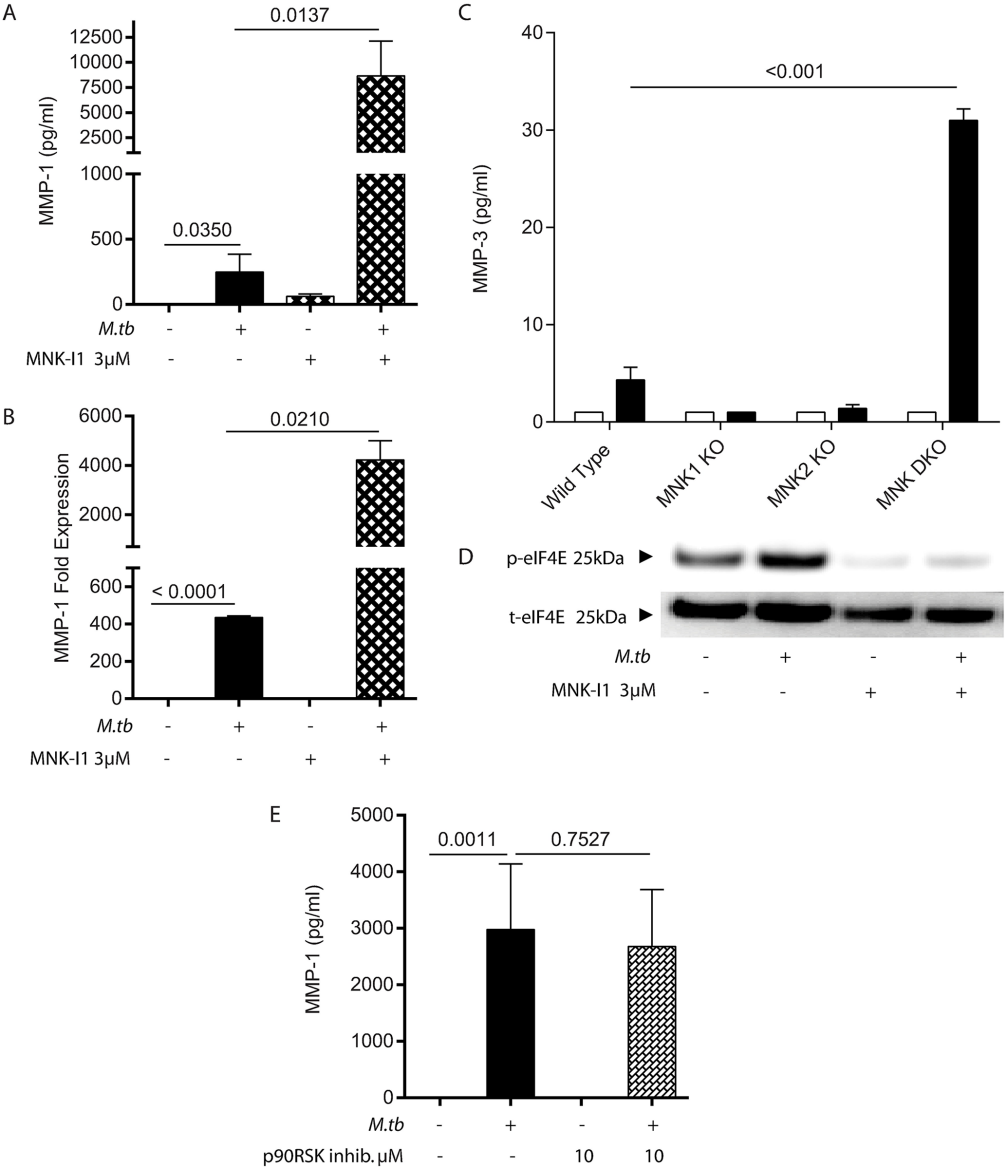
The MNK pathway is also a negative regulator of MMP-1 expression. **A.** Chemical inhibition of the MNK pathway by MNK-I1 significantly increases MMP-1 secretion from macrophages at 72h. **B.** Increased MMP-1 secretion is secondary to increased mRNA accumulation at 24h. **C.** Bone marrow derived macrophages from wild type, MNK1-KO, MNK2-KO or double KO mice were uninfected or infected with Mtb at MOI 1. Supernatants were harvested at 24h, sterile filtered and MMP-3 concentration analyzed by Luminex array. MMP-3 secretion was significantly elevated in the MNK double KO cells. Representative experiment of two each performed in triplicate. **D.** MNK inhibition suppresses phosphorylation of eIF4E at 180 mins. **E.** Inhibition of the p90^RSK^ pathway does not significantly change MMP-1 secretion at 72h. A, B: data represent experiments performed in triplicate on a minimum of two occasions, D: 3 separate donors, E: two donors in triplicate. Mean and standard deviation are shown and p values are Student’s t-test.

Next, we investigated the MNK pathway, which is downstream of p38 MAPK and regulates mRNA translation. We hypothesised that MNK inhibition would suppress MMP-1 production, but surprisingly MNK inhibition significantly augmented Mtb-driven MMP-1 secretion (Fig 5A). The increased secretion was secondary to increased MMP-1 gene expression (Fig 5B). To confirm that this is indeed an effect of disabling MNK function, we studied bone marrow-derived macrophages from mice in which the genes encoding MNK1 and/or MNK2 (termed *Mknk1* and *Mknk2*) have been disrupted [19]. Mice lack an orthologue of MMP-1 [20], and so we analyzed MMP-3 secretion, which is regulated in a very similar manner to MMP-1 [21]. Mtb infection increased MMP-3 secretion by murine wild type macrophages, and MMP-3 secretion was markedly higher in the MNK double knock-out cells, confirming that the effect of the MNK inhibitor does indeed reflect a negative input from the MNKs to MMP-3 secretion (Fig 5C). To confirm the efficacy of the MNK-I1 inhibitor [22], we performed Western blotting for phosphorylated eIF4E, a component of the eIF4F translation complex which is specifically phosphorylated by the MNKs and the only validated *in vivo* substrate. MNK inhibition suppressed eIF4E phosphorylation as expected (Fig 5D and S6 Fig). To determine if this effect involved the p90RSK pathway, which is downstream of ERK MAPK, we specifically inhibited this with BI-D1870 and found no change in MMP-1 secretion (Fig 5E), thereby demonstrating the crosstalk was not via this pathway.

**Fig 6:**
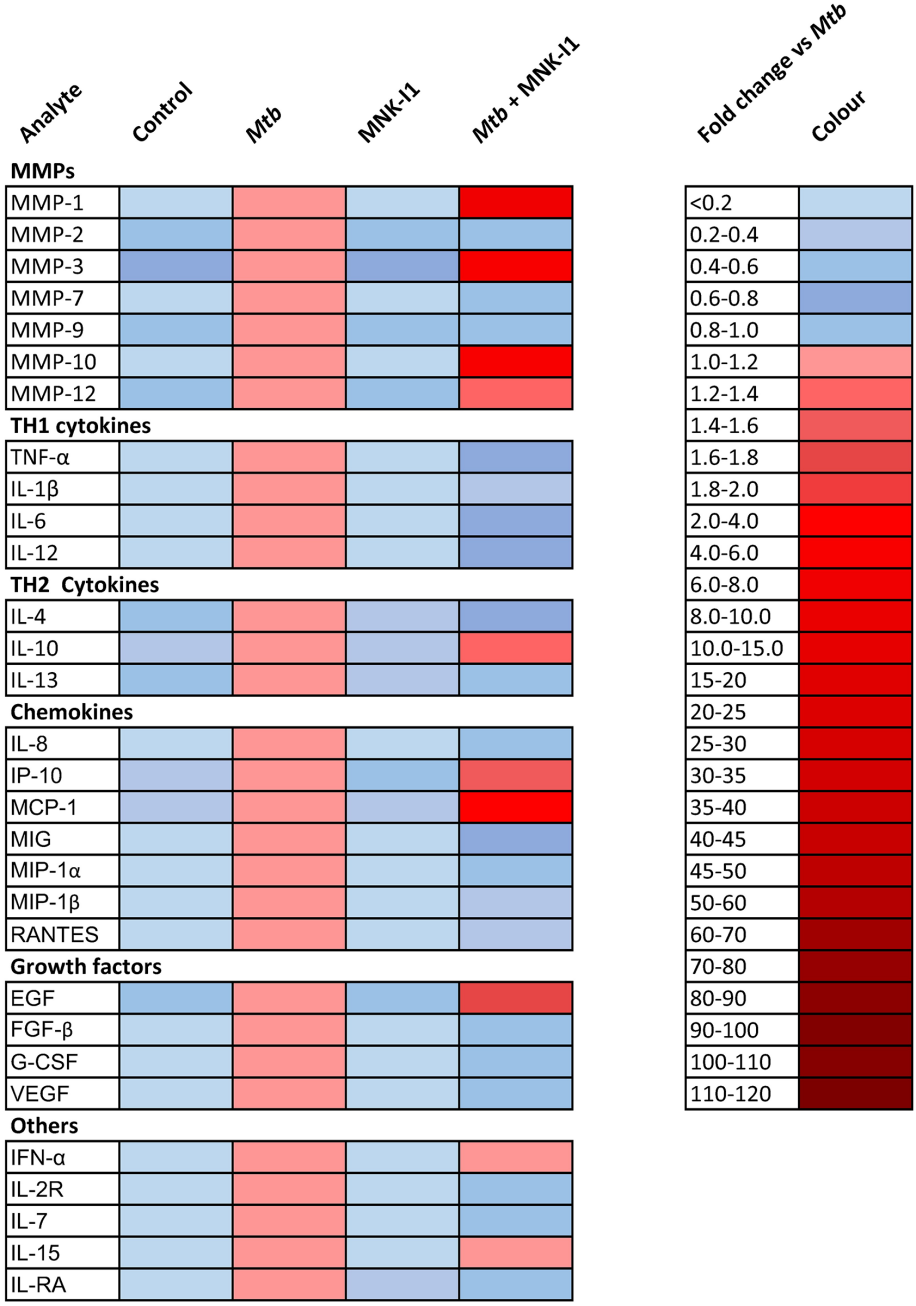
Negative regulation of MMPs by MNK is more specific than PI3K/AKT/mTORC1 signalling. Global analysis of MMP, cytokine, chemokine and growth factors secretion from macrophages at 72h by Luminex array demonstrates that MNK inhibition augments MMP-1, MMP-3 and MMP-10 secretion significantly, but conversely suppresses TH_1_, TH_2_ and chemokine secretion. Median values normalised to Mtb values of an experiment performed in triplicate are shown, and are representative of an experiment performed on two occasions.

To determine whether the negative regulatory effect of the MNKs was global, as we had observed for the PI3K/AKT/mTORC1 axis, or more specific, we performed luminex profiling of MMPs and cytokines. We demonstrated that the MNK effect was relatively specific to MMP-1, 3 and 10, and only significantly upregulated MCP-1 and EGF amongst the other inflammatory mediators studied (Fig 6). Blockade of the MNK pathway suppressed secretion of the majority of TH_1_ and TH_2_ cytokines, whereas they had been augmented by PI3K/mTORC1 pathway inhibition.

### PI3K kinase and MNK signalling intersect at eIF4E phosphorylation

**Fig 7:**
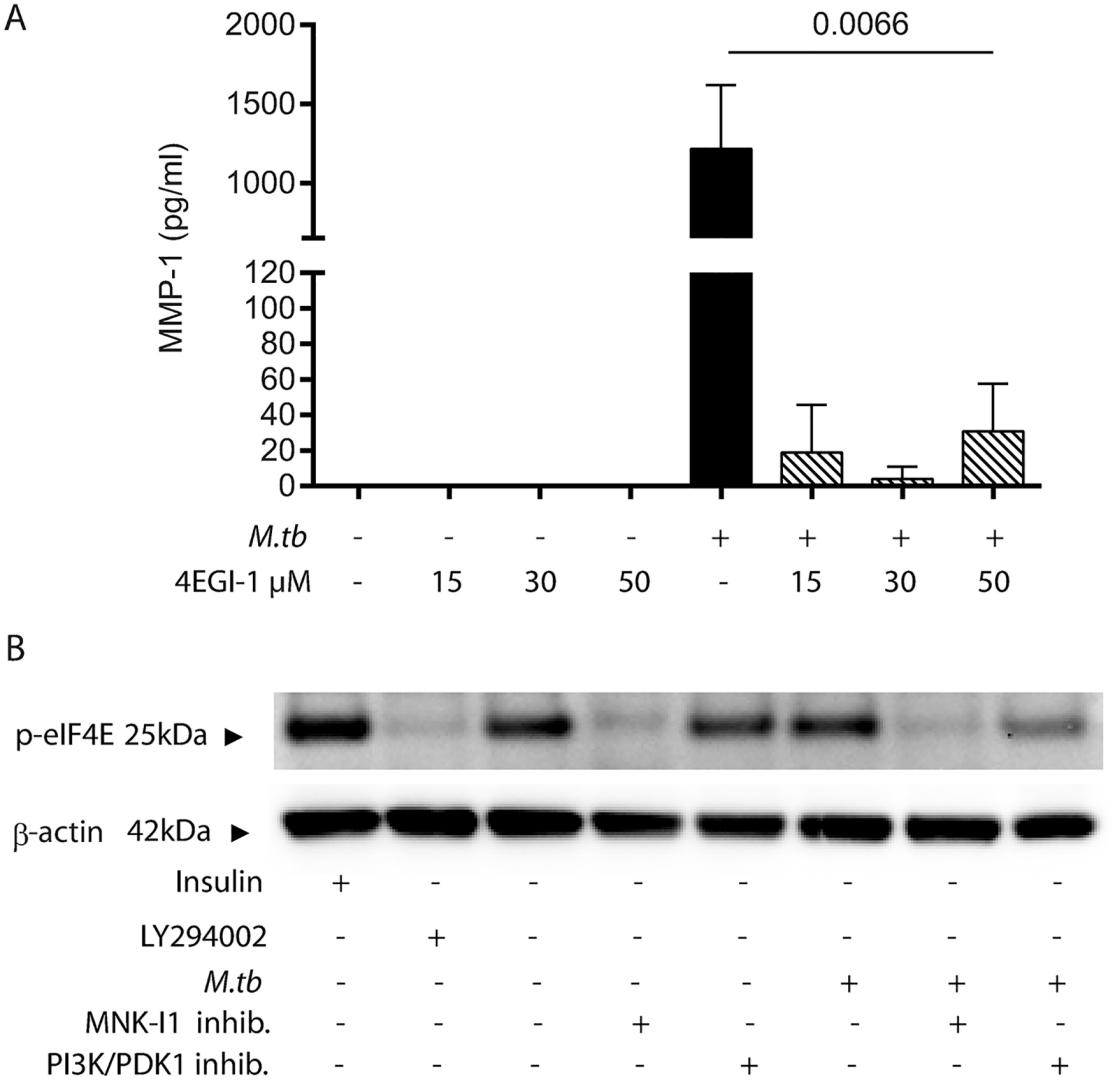
The PI3K and MNK pathways converge at the level of eIF4E phosphorylation. **A.** Inhibition of eIF4F complex formation significantly suppresses MMP-1 secretion from Mtb-infected macrophages at 72h. **B.** Insulin, a PI3K pathway activator, increases macrophage eIF4E phosphorylation, while LY294002 abrogates phosphorylation. Mtb infection does not change basal eIF4E phosphorylation levels, but either MNK or PI3K/PD-1 inhibition suppresses eIF4E phosphorylation at 90 mins. A: representative data from an experiment performed in 2 donors in triplicate, B: performed on three occasions in different donors. Mean and standard deviation shown and p values are by Student’s t-test.

We sought to identify a point where the negative regulatory pathways mediated by PI3K and MNK converge. First, we studied the effect of inhibition of the eIF4F translation initiation complex, which is required for 5’-cap-dependent protein synthesis. Inhibition of the translation complex, by disrupting the eIF4E and eIF4G interaction with 4EGI-1 [23], significantly suppressed MMP-1 secretion (Fig 7A), demonstrating that disrupting the translation factor complex suppressed synthesis as expected. We therefore then studied eIF4E phosphorylation. The positive control, insulin, an agonist of the PI3K pathway, increased eIF4E phosphorylation (Fig 7B, lane one and S6 Fig), whereas inhibition with LY294002, in the absence of any stimuli, inhibited eIF4E phosphorylation (lane two). MNK inhibition both without and with Mtb completely suppressed eIF4E phosphorylation as expected. In the context of Mtb stimulation, specific inhibition of PI3K/PDK1 reduced eIF4E phosphorylation (lane 8), providing evidence of convergence between MNK and PI3Kδ signalling at this level.

### Mtb upregulates a network of microRNAs with targeting sequences in the 3'UTRs of *PIK3CD*, *MLST8* and *MKNK1* mRNAs

**Fig 8:**
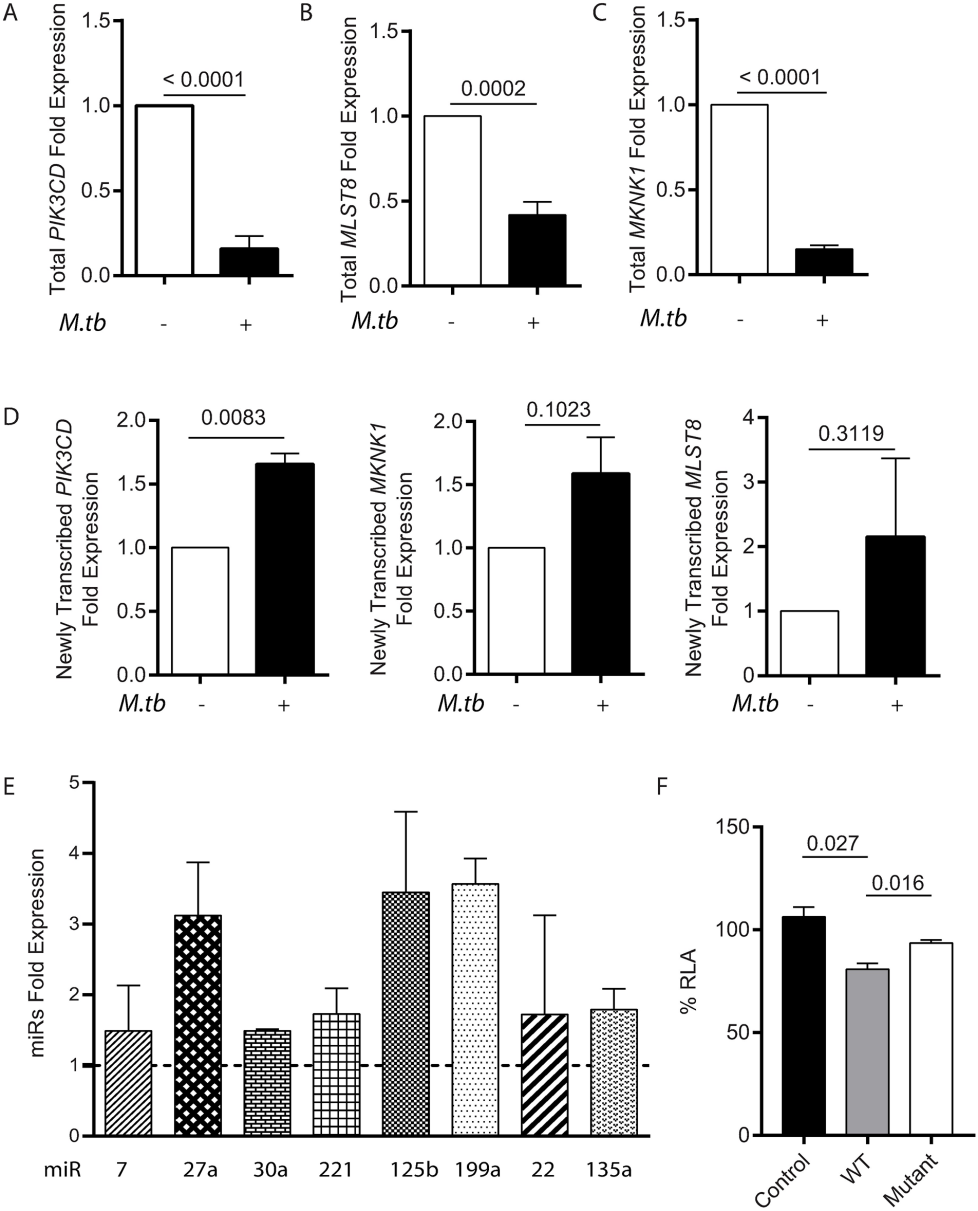
Mtb infection reduces stability of negative regulatory pathway genes and increases microRNA accumulation. **A, B, C.** Mtb infection of macrophages suppresses total *PIK3CD*, *MLST8* and *MKNK1* mRNA levels at 24h after infection. **D.** In contrast, synthesis of newly transcribed mRNA for each gene is increased at 24h. **E.** Mtb infection of macrophages upregulates multiple microRNAs that are predicted to target the negative regulatory pathways analyzed at 48h. **F.** miR-7 targeting the 3’UTR of *PIK3CD* suppresses luciferase activity. Hela cells were co-transfected with a miR-7 expressing vector (miR-7) and a Renilla Luciferase construct harbouring either a *PIK3CD*-3’UTR fragment containing the miR-7-5p binding site (bp 268–275) (Wild Type, WT), the same *PIK3CD*-3’UTR construct with mutated miR-7-5p binding site (bp 268–275) (Mutant), or empty construct (Control). RLA: Relative Luciferase Activity. Mean and standard deviation are shown, p values are by Student’s t-test and data are representative of experiments performed on at least two occasions in triplicate. F. Combined results from 4 independent experiments.

Finally, to investigate the mechanism whereby Mtb suppresses PI3Kδ expression, we studied mRNA stability within macrophages. First, we analyzed total cellular mRNA and demonstrated that Mtb not only suppressed *PIK3CD* in macrophages (Fig 8A), but also significantly suppressed expression of *MLST8*, a key subunit of mTORC1, and the MNK1 gene, *MKNK1* (Fig 8B and 8C). To determine whether this suppression was specific to Mtb, we studied different microbial stimuli. Mtb, TLR-2 stimulation and zymosan, a fungal wall component, each suppressed *MKNK1* gene expression in macrophages, while LPS and purified mycolic acid did not (S7 Fig).

To investigate the underlying mechanism, we performed mRNA pulldown experiments to characterise newly transcribed gene expression following Mtb infection. 4-Thio uridine was used to label mRNA and determine the ratio of newly-transcribed mRNA with total mRNA levels, and thereby demonstrate whether changes were due to altered synthesis or stability. Newly-transcribed *PIK3CD* mRNA expression was increased in infected cells, and *MLST8* and *MKNK1* were not suppressed (Fig 8D), suggesting the significantly reduced total mRNA levels were secondary to reduced mRNA stability. This suggested a role for post-transcriptional regulation via microRNAs increasing mRNA degradation, and we therefore analyzed microRNAs predicted by bioinformatic approaches to target these three mRNAs, using miRBase v21. Mtb infection upregulated multiple microRNAs, including miR27a, miR125b and miR199, all of which are predicted suppressors of the *PIK3CD*, *MLST8* and *MKNK1* mRNAs (Fig 8E). To confirm that *PIK3CD* could be targeted by these miR’s, we generated reporter constructs comprising the 3’-UTR fused to Renilla-luciferase. Transfection of HeLa cells with a plasmid expressing miR7, which is predicted to target *PIK3CD*, suppressed luminescence, whilst a site-directed mutant within the predicted binding region did not, confirming that miR-7 may directly bind to *PIK3CD* to reduce mRNA or protein levels (Fig 8F). Therefore, the reduced stability of mRNA is most likely secondary to Mtb-induced upregulation of microRNAs that target these transcripts.

## Discussion

**Fig 9:**
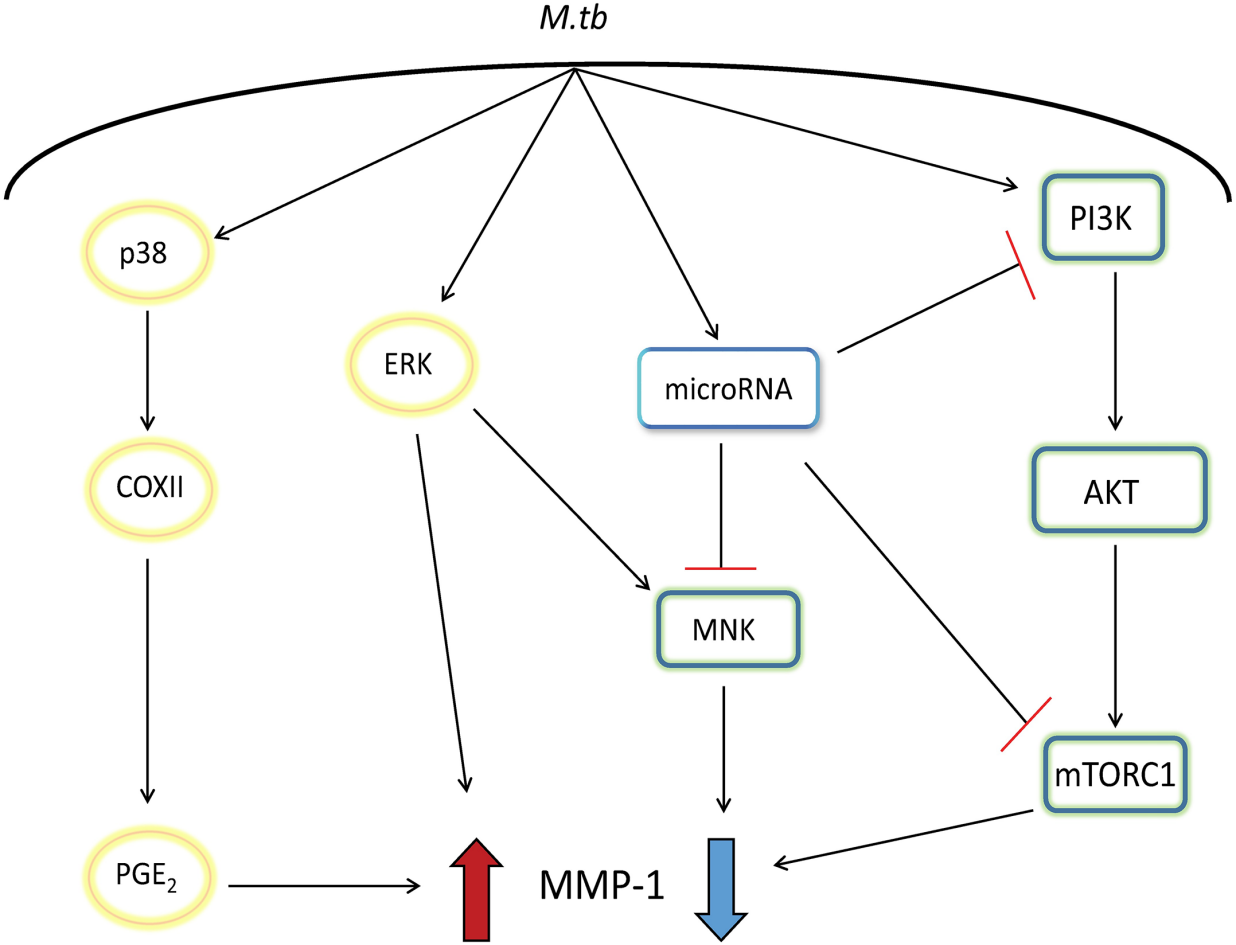
Mtb inhibits negative regulatory pathways in macrophages. Mtb upregulates MMP-1 via the MAPK pathways, whilst concurrently inducing microRNAs that target the negative regulatory pathways that limit MMP-1 expression, leading to excessive protease production and tissue destruction.

Mtb must cause pathology to be transmitted to new hosts, and patients with pulmonary cavities are the most infectious [3, 24]. Whilst the mechanisms whereby Mtb evades the host immune responses have been extensively investigated [25], relatively little is known about how Mtb engages the immune response to drive tissue destruction, cavitation and transmission [26]. Immune evasion will only lead to latent TB without onward transmission, and so the initiation of immunopathology is an essential event in the Mtb life cycle [27]. We have identified negative regulatory pathways that limit pathogenic MMP-1 secretion by primary human macrophages and observed the absence of PI3Kδ in TB granulomas in patients. We demonstrated that Mtb disables the PI3Kδ/AKT/mTORC1 and MNK regulatory pathways to drive a pro-tissue destructive phenotype, thereby uncovering a previously unidentified role of the MNK pathway as inhibitor of MMP-1 expression (Fig 9).

Direct macrophage infection by Mtb was required *in vitro* for suppression of *PIK3CD*, while widespread suppression was observed in lung granulomas, where mycobacteria are relatively sparse [28]. This suggests that either accumulation of Mtb antigens within granulomas may suppress *PIK3CD* expression in non-infected cells, or that a more complex regulation occurs during the long host-pathogen interaction in patients relative to the short-term cellular experiments possible *in vitro*. Since intercellular networks can upregulate MMP-1 secretion without suppressing *PIK3CD*, we propose that the down-regulation of the negative regulatory pathways serves to further augment a tissue-destructive proteolytic pathway caused by both direct infection and intercellular signalling to facilitate cavitation and transmission. Interferon-γ, a key cytokine in the host immune response to TB, also targets mTORC1 and MNK signalling [29], demonstrating the complex interplay between pathways and consistent with the emerging hypothesis that either an insufficient or excessive host immune response may be deleterious [30].

Pathology in TB results from dysregulation of inflammation [31] and MMPs are emerging as key pathological mediators [4]. p38 and ERK MAPKs are positive regulators of MMP expression, and p38 is phosphorylated in patients [8], while PI3K is a negative regulator in stromal cells [32]. We demonstrate in primary human macrophages that PI3K, AKT, mTORC1 and MNKs are all negative regulatory pathways, and are suppressed by Mtb infection. Consistent with our findings, PI3K limits IL-12 secretion and TLR signalling in LPS-stimulated dendritic cells [10, 11], suggesting that these early signalling events that occur immediately after receptor activation having a broad regulatory effect to diverse stimuli and lead to increased cytokine secretion. A similar role for PI3Kγ in controlling a macrophage switch between immune stimulation and suppression in cancer has very recently been described in murine macrophages [33] In that study, the signalling was via NFκB, but we were unable to demonstrate cross-talk at this level. Similar to our results in primary macrophages, in human PBMCs, Mtb has been shown to phosphorylate AKT and mTORC1, and rapamycin increases secretion of TNF-α, IL-1β and IL-6 [34]. The skewing of intracellular signalling in macrophages by Mtb may negate the effect of TH_2_ cytokines, such as IL-4 and IL-10, and regulatory T cells that are thought to limit immune-mediated tissue destruction in TB [30, 35].

We have identified a novel role for MNK signalling in limiting tissue-destructive MMPs and found that this was relatively specific, whereas the effect of PI3K/AKT/mTORC signalling on inflammatory mediators was much more widespread. The effect of MNK inhibition was striking and more pronounced that phenotypes observed with stimuli other than Mtb. MNK is downstream of p38 and ERK MAPK signalling [13], and is considered to regulates protein synthesis [36]. Therefore, we had predicted that MNK inhibition would suppress MMP-1. MMP-1 downregulation was observed when we directly suppressed eIF4F complex formation, whereas MNK inhibition increased MMP secretion while suppressing cytokines. We confirmed that the MNK inhibitor, MNK-I1, was acting specifically by blocking MNK function by studying MNK-deficient mouse cells. The majority of cytokines were suppressed by MNK inhibition, and so the overall effect may be to increase destruction of the extracellular matrix in the absence of an increased host inflammatory response that might favour bacterial killing. We demonstrated that other, but not all, infectious stimuli suppress *MKNK* gene expression, suggesting that this response may occur to a range of pathogen-derived molecules, and is usurped by Mtb to increase matrix destruction. This phenomenon requires further systematic dissection for full characterisation. MNK is thought to increase translation [12, 37], and therefore the phenotype of increased MMP-1 after MNK inhibition may be due to reduced inhibitory transcription factor production, as has been demonstrated for type I interferons in viral infection [38]. Therefore, our findings suggest that modulating MNK will exert a more nuanced effect within the cellular machinery than purely inhibiting protein synthesis.

Our RNA analysis suggested Mtb infection reduced stability of mRNAs encoding negative regulatory proteins, and we identified Mtb-driven increases in microRNAs that target the signalling cascade at multiple levels. We demonstrated that miR-7 binds to the 3’UTR of *PIK3CD*, as predicted bioinformatically. miRNAs are responsible for fine tuning transcription and usually multiple miRNAs combine to reduce the levels of a given mRNA with an additive effect [15]. We identified that multiple miRNAs predicted to target each negative regulatory pathway [39] were increased in Mtb infection, suggesting an overall effect skewing the transcriptional response to a matrix-degradative phenotype. However, systematic characterisation of each miR interaction with each regulatory pathway will be required to fully confirm this hypothesis. We demonstrated that intercellular networks could augment MMP-1 secretion, but did not suppress *PIK3CD* gene expression, indicating that dual regulatory effects are likely to be operant *in vivo*, with infected cells being particularly predisposed to excessive protease secretion. Cytokine networks may augment MMP secretion by bystander cells in the absence of *PIK3CD* suppression. Since mice do not express MMP-1 [40], further dissection of this regulatory pathway will likely require gene editing in advanced human cell culture model systems.

In summary, we identify a novel strategy employed by Mtb to drive immunopathology by disrupting negative regulatory pathways, both in primary macrophages and in patients. We demonstrate a previously unrecognised role of the MNK pathway as a negative regulator of MMP secretion. Inhibitors of PI3Kδ are already in clinical use, and other drugs are in development, and therefore it is possible that they may increase the risk of active TB in the same way as anti-TNF-α agents [41]. Furthermore, novel host-directed therapies that target intracellular signalling pathways to enhance Mtb killing [42] must not inadvertently suppress the negative regulatory pathways and thereby augment immunopathology.

## Materials and methods

### Ethics statement

Samples used in this study were sourced from the Southampton Research Biorepository, University Hospital Southampton NHS Foundation Trust and University of Southampton, Mailpoint 218, Tremona Road, Southampton, SO16 6YD. Lung biopsy tissue was taken as part of routine clinical care and tissue blocks excess to diagnostic testing were analyzed in this study. The project was approved by the Institutional Review Board (Reference 12/NW/0794 SRB04_14). The ethics committee approved the analysis of this tissue without individual informed consent since it was surplus archived tissue taken as part of routine care. For analysis of blood from healthy donors, this work was approved by the National Research Ethics Service committee South Central—Southampton A (ref 13 SC 0043) and all donors gave written informed consent.

### Chemicals and reagents

Standard laboratory reagents were from Sigma Aldrich. Chemical inhibitors were pan-PI3K: LY294002; PI3Kδ: IC87114; mTORC1: Rapamycin (Merck Millipore); AKT: MK-2206 (Insight Biotechnology); PI3K/PDK-1: NVP-BAG956; eIF4E/eIF4G interaction: 4EGI-1 (Merck Chemicals); P90RSK: BI-D1870 (Selleckchem). MNK-I1 was kindly synthesised by Professor Jiang Tao and her colleagues at the Ocean University of China, Qingdao, China. Infectious stimuli were: Zymosan (Sigma, 100μg/ml), LPS (Sigma, 1μg/ml), TLR-2 agonist Pam Cys-Ser-(Lys) [Pam3Cys] (MerkMillipore, 100ng/ml), Mycolic acid (Sigma, 10 μg/ml).

### PBMC cell isolation from human blood

PBMCs were isolated from single donor leukocyte cones (National Health Service Blood and Transfusion, Southampton, UK) or fresh blood from healthy donors by density gradient centrifugation over Ficoll-Paque (GE Healthcare Life Sciences). Monocytes were plated at 250,000cm^2^, adhered for 1 hour and then washed 3 times to remove non-adherent cells. Monocytes were matured to macrophages for 4 days in complete RPMI with 10% human serum with 100ng M-CSF, then rested for 1 day in complete medium without growth factors, and then the experiment was started (Day 0).

### *M*. *tuberculosis* culture

*M*. *tuberculosis* H37Rv (Mtb) was cultured in Middlebrook 7H9 medium (supplemented with 10% ADC, 0.2% glycerol and 0.02% Tween 80) (BD Biosciences, Oxford) with agitation. Cultures at 1x 10^8^ CFU/ml Mtb (OD = 0.6) was used for all experiments.

### Macrophage infection

Macrophages were infected with Mtb at MOI of 1. Cells were washed one hour after infection to remove non-phagocytized Mtb. Experimental duration was between hours for analysis of protein phosphorylation and 3 days for secretion. For experiments involving chemical inhibitors, cells were pre-incubated with inhibitor for 2h prior to infection with Mtb.

### MMP-1 ELISA and Luminex array

MMP-1 concentrations were analyzed by ELISA assay according to manufacturer’s protocol (R & D Systems). For multianalyte profiling, MMP and cytokine concentrations in cell culture supernatants harvested at 72h were analyzed on a Bioplex 200 platform (Bio-Rad, Hemel Hempstead, U.K.). MMP concentrations were analyzed by the MMP Fluorokine multianalyte profiling (R&D Systems, Abingdon, U.K) and cytokine concentrations were measured using the Cytokine Human 30-Plex Panel for the Luminex platform (Invitrogen, Paisley, UK) according to manufacturers’ protocol.

### Phosphorylation Western blotting

MDMs were infected with Mtb and lysed with 200μl SDS sample buffer (62.5mM Tris pH 6.8, 2% SDS, 10% glycerol, 50mM DTT, 0.01% Bromophenol blue) at defined time points. Samples were filtered through 0.2μM Anopore filter and frozen at -80°C. 20μl aliquots were heat denatured and run on a 10% acrylamide gel at 200V (Running buffer 25mM Tris base, 192mM glycine, 0.1% SDS) for 3h. Gels were electro-transferred to a nitrocellulose membrane (Amersham) and blocked for 1h with 5% milk protein / 0.1% Tween-20. The membrane was incubated with primary antibody (AKT or phospho-AKT Ser 473, p38 or phospho-p38, ERK or phospho-ERK, Cell Signalling Technology; eIF4E and phosphor eIF4E, Merck Millipore) in 5% BSA / 0.1% Tween at 4°C overnight. Blots were then washed three times and incubated with HRP-linked anti-rabbit secondary antibody (Cell Signalling Technology, 1/2000 dilution in 5% milk protein / 0.1% Tween) for 1h. Luminescence was detected with the ECL system (Amersham) according to manufacturer’s protocol. Immunoblotting for total p38, ERK or AKT confirmed equal loading between samples.

### Quantitative RT-PCR

MDMs were lysed with TRI-reagent (Sigma-Aldrich), isolated with chloroform phase separation and precipitated with isopropanol. RNA was quantified by NanoDrop and retro-transcribed to complementary DNA by the High Capacity cDNA Reverse Transcription kit (Life Technologies, Paisley, UK). cDNAs obtained were then used for micro RNA and gene expression quantification assays by RT-qPCR for *MMP-1* (Hs00899658_m1), *PIK3CD* (Hs00192399_m1), *MLST8* (Hs00909882_g1), *MKNK1* (Hs00374375_m1) and *GAPDH* (Hs02758991_g1) following manufacturer's instruction (Applied Biosystems, USA). For microRNA’s, miRNA primers were from TaqMan OpenArray Human MicroRNA Plate (ThermoFisher Scientific, UK), with the following catalogue numbers: miR-7 TM000268, miR-27a TM000408, miR-30a TM000417, miR-221 RT524, miR-125b TM449, miR-199 TM498, miR-22 TM000398, miR-135a TM000480. Taqman Universal master mix and primers specific for the gene of study and *GAPDH* as house-keeping gene were used. Each RT-qPCR experiment was performed in duplicates and results were analyzed using SDS version 2.3 sequence detection systems (Applied Biosystems, USA). Comparative CT method was employed to analyze all RT-qPCR data.

### Immunohistochemistry

Paraffin-embedded lung tissues were mounted at 4μm thin onto APS coated glass slides and dried. Sections were dewaxed and 30% hydrogen peroxide used to block endogenous peroxidase. Sections were washed three times in TBS buffer and heat induced-epitope retrieval employed by boiling the slides in 1mM EDTA (pH 8.0) in distilled water for 25 minutes. Slides were incubated in Avidin solution for 20 minutes, followed by 3 washes. This was followed by incubation in biotin solution for 20 minutes followed by another wash step. The slide was blocked with Dulbecco’s Modified Eagle Medium (DMEM) containing with 10% FCS and 2% BSA for 20 minutes. Slides were incubated at 4°C overnight in appropriately diluted primary antibody; 1:1000 dilution of anti-PIK3δ (LifeSpan BioScience,Inc) or ready–to-use monoclonal mouse CD68 antibody, clone PG-M1 (Dako, IR613). Sections were washed and incubated in 1:400 of biotinylated rabbit anti mouse (Dako) secondary antibody for 30 minutes. After a second wash, sections were incubated for 30 minutes in streptavidin biotin-peroxidase complexes (Elite vectastain ABC kit, Vector laboratories). Sections were washed and incubated in DAB (2-component DAB pack, BioGenex) substrate for 5 minutes. Counter staining was performed in Mayer’s haematoxylin for 20 seconds. Dehydration of slides was performed at 1 minute in graded alcohols and mounted in pertex. Images were captured on Olympus CC12 (dotSlide) microscope.

### COX-2 flow cytometry analysis

MDMs were infected with Mtb, in the presence or absence of inhibitors, as above. Cells were fixed in 4% paraformaldehyde for 30 minutes at room temperature then lifted mechanically. Anti-COX II FITC labelled intracellular staining was performed according to manufacturer’s instructions (Cayman Chemicals). Briefly, cells were washed and permeabilized with 0.5% BSA, 0.1% Na azide, 0.1% Saponin solution. After a further wash step, cells were incubated with primary antibody (1/10 dilution) or IgG control (1/200 dilution, mouse IgG1 FITC, Serotec) for 30 minutes at room temperature in the dark. Cells were re-suspended in PBS and analyzed by flow cytometry on a Becton Dickinson FACS Calibur.

### Nuclear and cytoplasmic extract preparation

MDMs were infected with Mtb, in the presence or absence of inhibitors and cytoplasmic and nuclear extracts were prepared using the NE-PER kit (Pierce Biotechnology, Perbio) according manufacturer’s instructions. Briefly, cells were mechanically lifted into ice cold PBS, microcentrifuged and the supernatants removed leaving the pellet as dry as possible. Halt Protease Inhibitor Cocktail (Pierce, Perbio) was added to the stock solutions and the appropriate volume of ice cold CERI was added to the pellet. After re-suspension by vortexing, samples were incubated on ice for 10 minutes and CERII solution was added. Samples were vortexed and after centrifugation at 4°C for 5 minutes the supernatant containing the cytoplasmic extract was immediately collected, and filtered through a 0.2μM Durapore filter (Millipore). Ice cold NER was added to the remaining pellet containing nuclei, and vortexed for 15 seconds every 10 minutes for a total of 40 minutes on ice. After centrifugation the supernatant was harvested, filtered through a 0.2μM Durapore filter (Millipore) and all extracts stored at -80°C.

### TransAm ELISAs

Transcription factor activation in nuclear extracts was determined by TransAm ELISA based assay kits (Active Motif, UK) according to manufacturer’s instructions. Total protein concentration was measured by Bradford assay (Biorad) and 5μg of nuclear extract was used for each sample. The lower level of sensitivity was <0.5μg of nuclear or whole cell extract.

### Bone Marrow Derived Macrophages from MNK deficient mice and MMP-3 luminex

Bone Marrow Derived Macrophages (BMDM) from MNK1, MNK2 or MNK Double mutant mice were acquired from South Australian Health and Medical Research Institute, Australia. After defrosting, macrophages were cultured in complete macrophage medium, which is composed of L929 cell conditioned medium, 20% FCS and DMEM with 10ng/ml of M-CSF overnight, and were infected with Mtb H37Rv at MOI of 1 for 24 h. Supernatants were harvested for analysis and sterile filtered. Mouse MMP-3 Fluorokine beads (R&D Systems) were used to measure concentrations of MMP-3 in supernatant from BMDM on the Luminex 200 platform (Bio-Rad, Hertfordshire, UK). The lower limit of detection of the assay was 2 pg/ml. Assays were performed per manufacturer’s instructions.

### Thio uridine labelling of RNA and pulldown analysis

To analyze newly synthesised mRNA, 100μM of 4-Thio uridine was added to infected macrophages for at least 24h, lysed in Tri-reagent and total RNA extracted. 1mg/ml of EZ-Link biotin-HPDP (Pierce, Thermo Fisher Scientific UK Ltd.) solution was prepared in Dimethylformamide (DMF). 1μl of 1mg/ml of EZ-Link biotin-HPDP solution was used per μg of total 4-TU labelled RNA. Master mix was prepared from 1M Tris (pH 7.4) and 0.5M EDTA to a final concentration of 10mM Tris and 1mM EDTA, in RNase free water. Isopropanol at volume equal to the final reaction volume and 5M NaCl (at 1/10^th^ final reaction volume) were added to the biotinylated 4-TU labelled RNA. The mixture was vortexed and incubated at room temperature for 5 minutes before being centrifuged at 13000rpm for 20 minutes. The RNA pellet was washed in 250μl 75% Ethanol, followed by 10 minutes centrifugation. The pellet was allowed to air-dry until semi-transparent, and RNA was re-suspended in 20–50μl of RNase free water. The re-suspended RNA was purified immediately or stored at -80°C for future pulldown analysis.

To isolate and purify the 4-TU labelled RNA, Magnetic Porous Glass (MPG) streptavidin beads (Pure Biotech LLC) was used to bind and pull down the biotinylated 4-TU labelled RNA. The beads were incubated with tRNA (1μg per 5μl of beads) and rotated at room temperature for 20 minutes. Tubes were placed in magnetic stand beads for 1 minute. This was followed by three washes in 300μl of MPG buffer (1M NaCl, 10mM EDTA, 100mM Tris-HCL at pH 7.4 in RNase free water). Beads were re-suspended in MPG buffer equal to the original volume of beads. Volume of RNA and beads were adjusted to be equal, to allow 1:1 combination ratio. The biotinylated 4-TU RNA was added to the beads and incubated at room temperature with rotation for 1 h. Beads were collected in a magnetic stand for 1 minute. The supernatant was collected and kept as unbound, non-4TU RNA. Beads were washed two times in 250μl of room temperature MPG buffer, one wash in 65°C MPG buffer, and a final wash in 50μl MPG buffer. The supernatant from the last wash was kept as ‘wash RNA’ to check for flow through RNA.

To elute the bead-bound, 4-TU labelled RNA, freshly prepared 5% β-mercaptoethanol was added at volume equal to original bead volume and incubated at room temperature with rotation for 20 minutes. The tubes were centrifuged, and beads collected in a magnetic stand for 1 minute. The supernatant was kept as bound, 4-TU labelled RNA. RNA was precipitated as described above by adding 5M NaCl at 1/10^th^ the RNA volume, isopropanol at the same volume as the RNA, and 1μg glycogen. The mixture was incubated at room temperature for 5 minutes and centrifuged for 20 minutes. The RNA pellet was washed in 250μl 75% Ethanol followed by 10 minutes centrifugation. The pellet was allowed to air-dry until semi-transparent, and RNA was re-suspended in 20–50μl of RNase free water. The re-suspended RNA was placed in the magnetic stand to collect any residual beads. The supernatant was collected in newly labelled nuclease free tubes as purified, newly synthesised RNA samples.

Purified 4-TU labelled RNA was treated with rDNase (DNA-free Kit, Life Technologies) to remove potential genomic DNA contamination. SuperScript III Reverse Transcriptase (Invitrogen), Oligo (dt)12–18 primer (Invitrogen) and random hexamer (Component of high capacity cDNA kit, Life Technologies Ltd.) were used to retro transcribe 5μg of the pulled down RNA to cDNA in a 20μl reaction volume according to the manufacturer’s protocol. The mixture was heated at 65°C for 5 minutes, after which tubes were incubated on ice for a further 1 minute. 4μl, 1μl and 2μl of 5x first strand buffer, 0.1MDTT, SuperScript III Reverse Transcriptase respectively (all from Invitrogen) and 1μl of RNaseOUT (Invitrogen) were added. Reverse transcription was performed by incubating tubes at 25°C for 5 minutes, 55°C for 40 minutes and at 70°C for 15 minutes. The cDNA was stored in -20°C for RT-qPCR amplification as above.

### Micro-reverse transcription (micro-RT)

To quantify expression of the micro RNAs of interest, cellular RNA was converted to cDNA as above. For micro-RT, 10ng/μl of RNA concentration (diluted using RNase-free water) and stem-loop primers that are specific for each of the microRNA of interest were used. RT-qPCR was performed using TaqMan Gene Expression Assays (Thermo Fisher Scientific).

### Vector construction and transfection experiments

Regions of interest were first amplified from genomic DNA by PCR using GoTaq G2 Polymerase (Promega) with standard additions to the PCR mastermix. The genomic region encompassing miR-7-3 was amplified using the following forward; CTCGAGGGGTCTCAGACATGGGGCAGAGGG and reverse; AAGCTTCCACTGGCCAGCCCATTGAAGGCG primers with XHOI and HINDIII restriction sites. For the *PIK3CD*-3’UTR, forward; TCTAGACAAGCACATTGGTCCTAAAGGGGC and reverse; GCGGCCGCAAGGCATCCTGTCGGACAGTAGGC primers with XBAI & NOTI sites were used to amplify a 362nt fragment of the *PIK3CD*-3’UTR containing the 8mer miR-7-5p binding site. Each product was cloned separately into pCR 2.1-TOPO (Invitrogen) and amplified in plasmid DNA using the TOPO TA cloning kit method (Invitrogen) and chemically competent *E*. *Coli*. The amplified sequences of interest were removed from pCR 2.1 TOPO and inserted into pCDNA 3.1 (miR-7) or pRLTK (*PIK3CD*-3’UTR) using XHOI/HINDIII or XBAI/NOTI restriction sites, respectively.

To assess the specificity of the miR-7-5p putative binding site in the *PIK3CD*-3’UTR fragment, a 4nt substitution removing sequence complementarity to the miR-7-5p seed sequence was performed by QuickChange Site Directed Mutagenesis (Stratagene), using a previously outlined method [43]. Primer sequences for the *PIK3CD*-3’UTR mutant were forward; GGATTGTCACCCCAAGGATCCCAGCTGGTGGATCTG and reverse; CAGATCCACCAGCTGGGATCCTTGGGGTGACAATCC.

To determine direct targeting of *PIK3CD* by miR-7, the pCDNA 3.1_miR-7 construct was co-transfected in to HeLa cells with either pRL-TK_*PIK3CD*-3’UTR or pRL-TK_*PIK3CD*-3’UTR_MUTANT vectors, using SuperFect (Qiagen) and manufacturers recommendations. Empty pRL-TK vector and a pCDNA 3.1 construct containing a non-related insert (PU.1 3’UTR) were used as control vectors to elucidate miR-7 activity against *PIK3CD*-3’UTR. The pGL3 Luciferase reporter (Promega) was used as a normalising vector to assess transfection efficiency. Luminometry was performed using the Dual-Glo Luciferase assay system (Promega) following manufacturer’s instructions. Experimental conditions performed in duplicate were averaged, and 4 independent experiments were performed.

### Statistical analysis

Analysis was performed using Graphpad Prism v6.0. Multiple intervention experiments were compared with the One Way ANOVA followed by Tukey’s multiple comparison. A p value of <0.05 was taken as statistically significant. For secretion data, experiments were performed in triplicate on a minimum of 2 occasions, while for RNA analysis representative data from at least 3 independent experiments is shown.

### Accession numbers

Uniprot accession numbers for the principal proteins discussed are; MMP-1 P03956, PI3Kδ O00329, mTORC1 P42345, MNK Q9BUB5

## Supporting information

S1 Fig Expression of PIK3CD is not suppressed by PI3K pathway inhibitors. Primary macrophages were incubated with inhibitors for 24h and PIK3CD expression analyzed by RT-qPCR. PIK3CD expression in fact was increased after 24h of inhibitors. * p < 0.05, ** p < 0.01.
(TIF)

S2 Fig Densitometry of AKT, p38 and ERK western blots. Digital image analysis of western blots was performed using Image J and normalised to loading controls. **A**, **B**, **C**: AKT. **D**, **E**: p38. **F**, **G**: ERK.
(TIF)

S3 Fig Intercellular networks augment macrophage MMP-1 production. Conditioned media was prepared from uninfected or Mtb-infected macrophages that had been harvested at 72h after infection and sterile filtered. Macrophages were stimulated with this conditioned media at 1 in 5 dilution and incubated for 72h before analysis. MMP-1 concentrations in cell culture supernatant was analyzed by Luminex. Conditioned media from infected macrophages significantly increased MMP-1 secretion compared to media from uninfected macrophages, demonstrating intercellular networks augment MMP-1 secretion. Representative data of an experiment performed in triplicate on 2 occasions is shown.
(TIF)

S4 Fig CD68 is widely expressed in TB granulomas while PI3Kδ is absent. **A.** CD68 immunohistochemistry of lung biopsy at low power (x4). **B.** PI3Kδ expression is absent within the granuloma (x4). **C.** Tonsil positive control for CD68. **D.** Kidney positive control for PI3Kδ. **E.** Tonsil, secondary antibody alone. **F.** Kidney, secondary antibody alone.
(TIF)

S5 Fig mTORC1 inhibition has global effects on MMP, cytokine and chemokine secretion by macrophages. Luminex profiling of MMPs, cytokines, chemokines and growth factors demonstrates the majority of mediators upregulated by Mtb were further augmented when mTORC1 signalling was inhibited by rapamycin. Median values normalised to Mtb-infected samples of an experiment performed in triplicate are shown, and are representative of an experiment performed on two occasions.
(TIF)

S6 Fig Densitometry of eIF4E western blots. Digital images of western blots was performed using Image J and normalised to loading controls. **A**: eIF4E / p-eIF4E. **B**: eIF4E / β-actin
(TIF)

S7 Fig Mtb, fungal stimuli and TLR-2 stimulation suppress *MKNK1* gene expression but other infectious stimuli do not. Primary human macrophages were unstimulated or stimulated with Mtb (MOI 1), zymosan (100μg/ml), LPS (1μg/ml), TLR-2 agonist Pam3Cys (100ng/ml) or mycolic acid (10μg/ml). Cells were lysed at 48h with Trizol and *MKNK1* gene expression analyzed by RT-qPCR. Mtb, zymosan and TLR-2 stimulation suppressed *MKNK1* expression, while LPS and mycolic acids did not.
(TIF)
