# Supplementary figures and images for: Mycobacterium tuberculosis subverts negative regulatory pathways in human macrophages to drive immunopathology
Source: PLoS Pathog. 2017 Jun 1;13(6):e1006367. doi: 10.1371/journal.ppat.1006367 (PMC5453634; doi:10.1371/journal.ppat.1006367)

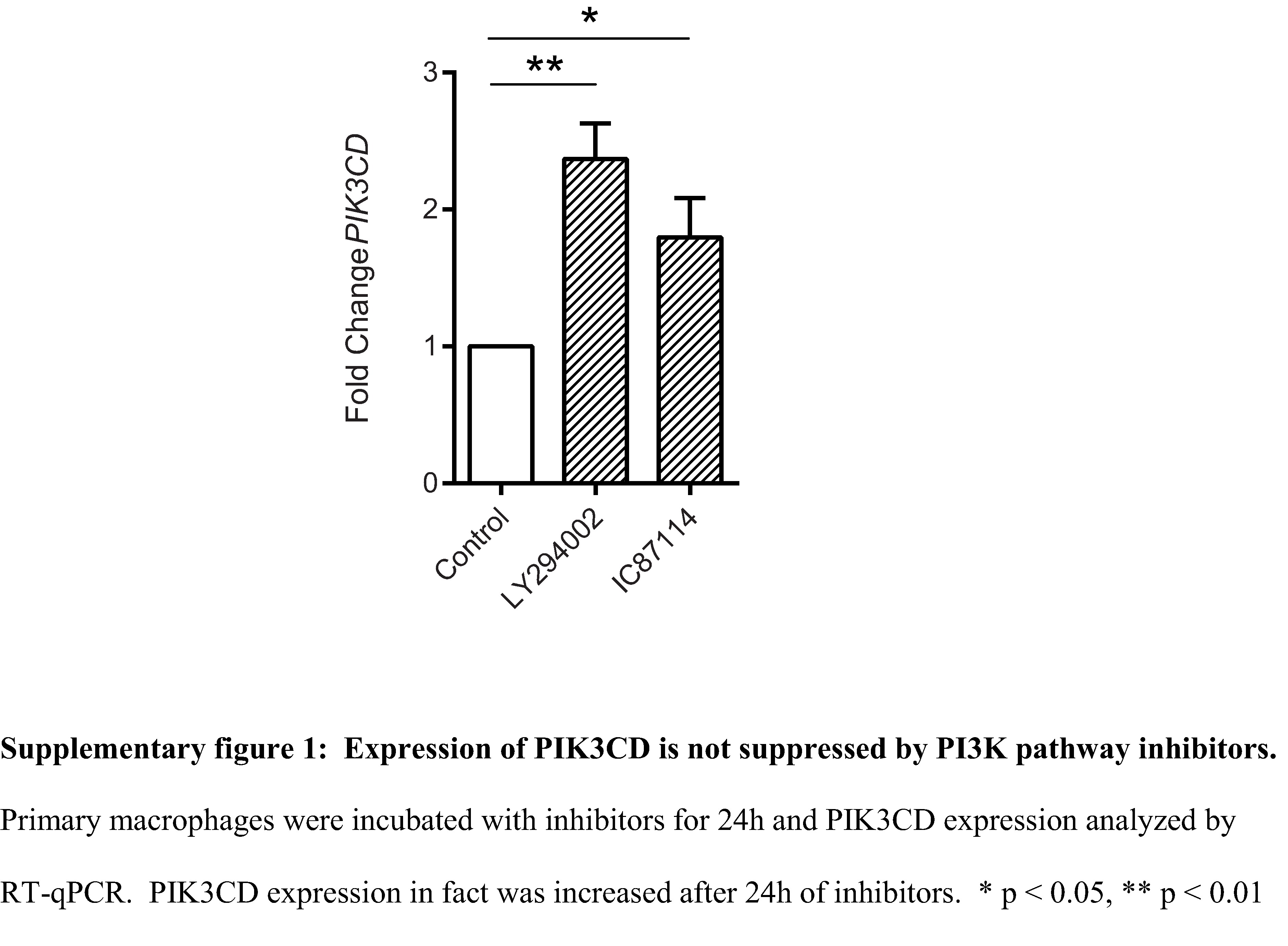

Supplement: S1 Fig — Primary macrophages were incubated with inhibitors for 24h and PIK3CD expression analyzed by RT-qPCR. PIK3CD expression in fact was increased after 24h of inhibitors. * p < 0.05, ** p < 0.01. (TIF) [file ppat.1006367.s001.tif]

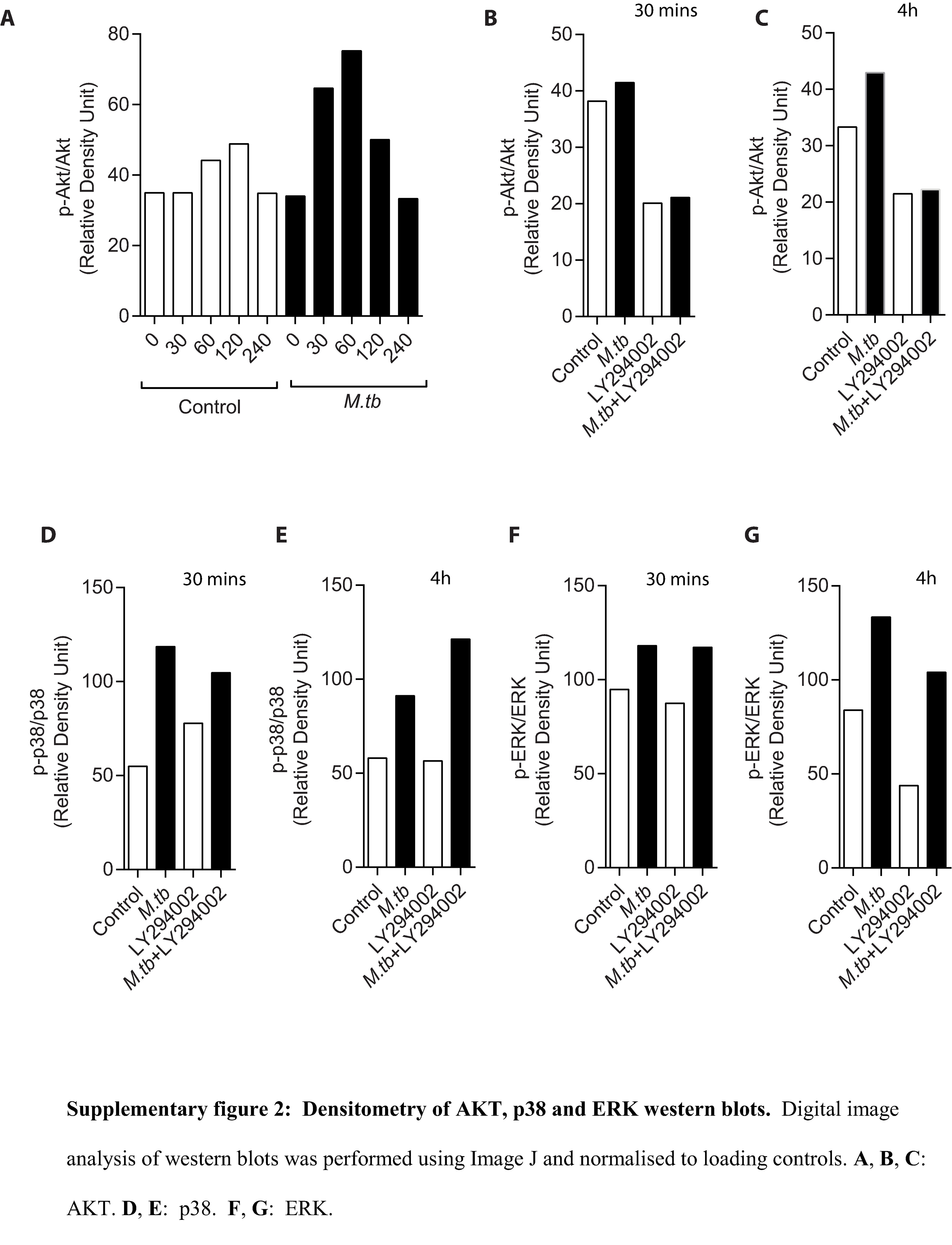

Supplement: S2 Fig — Digital image analysis of western blots was performed using Image J and normalised to loading controls. A, B, C: AKT. D, E: p38. F, G: ERK. (TIF) [file ppat.1006367.s002.tif]

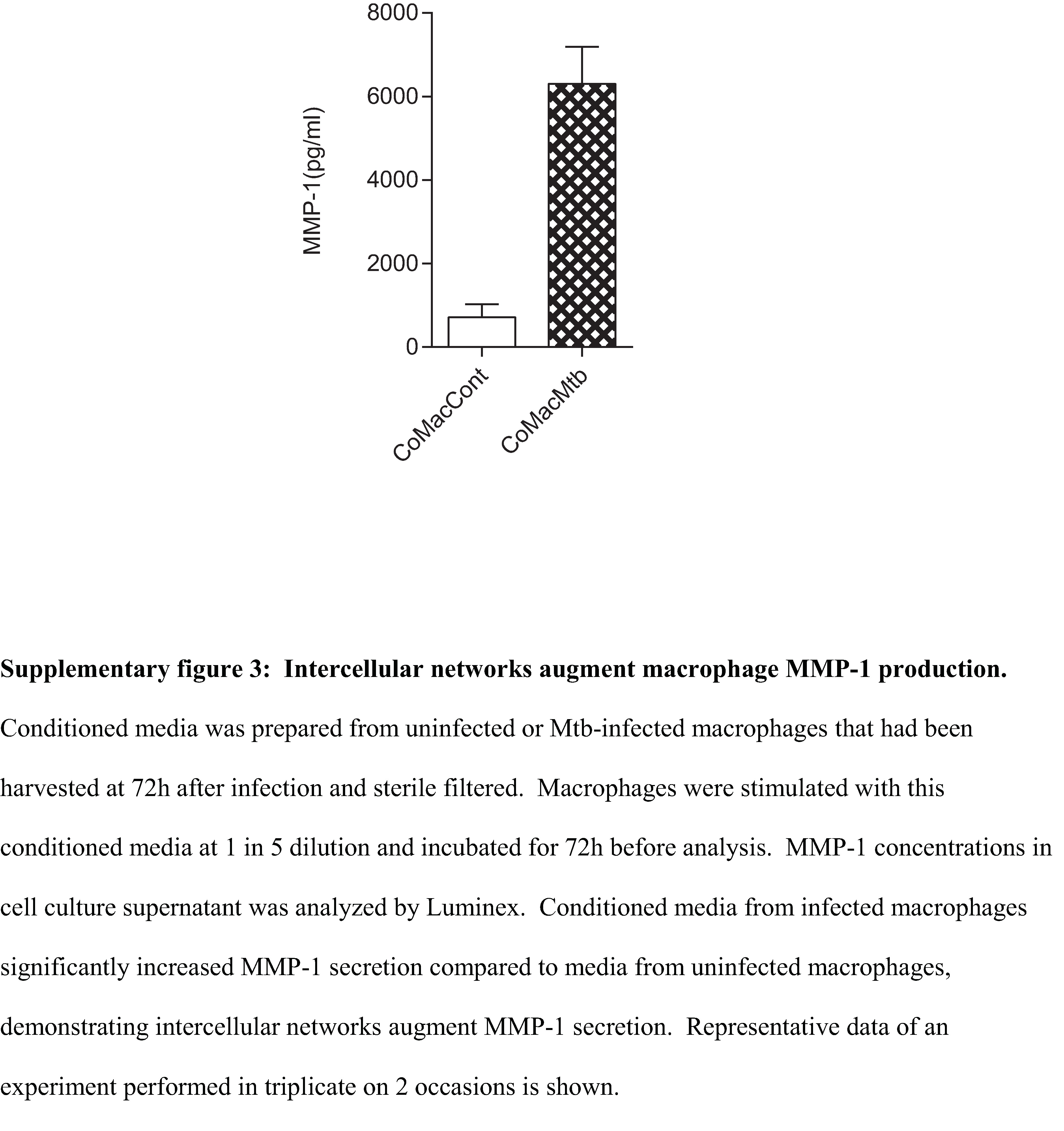

Supplement: S3 Fig — Conditioned media was prepared from uninfected or Mtb-infected macrophages that had been harvested at 72h after infection and sterile filtered. Macrophages were stimulated with this conditioned media at 1 in 5 dilution and incubated for 72h before analysis. MMP-1 concentrations in cell culture supernatant was analyzed by Luminex. Conditioned media from infected macrophages significantly increased MMP-1 secretion compared to media from uninfected macrophages, demonstrating intercellular networks augment MMP-1 secretion. Representative data of an experiment performed in triplicate on 2 occasions is shown. (TIF) [file ppat.1006367.s003.tif]

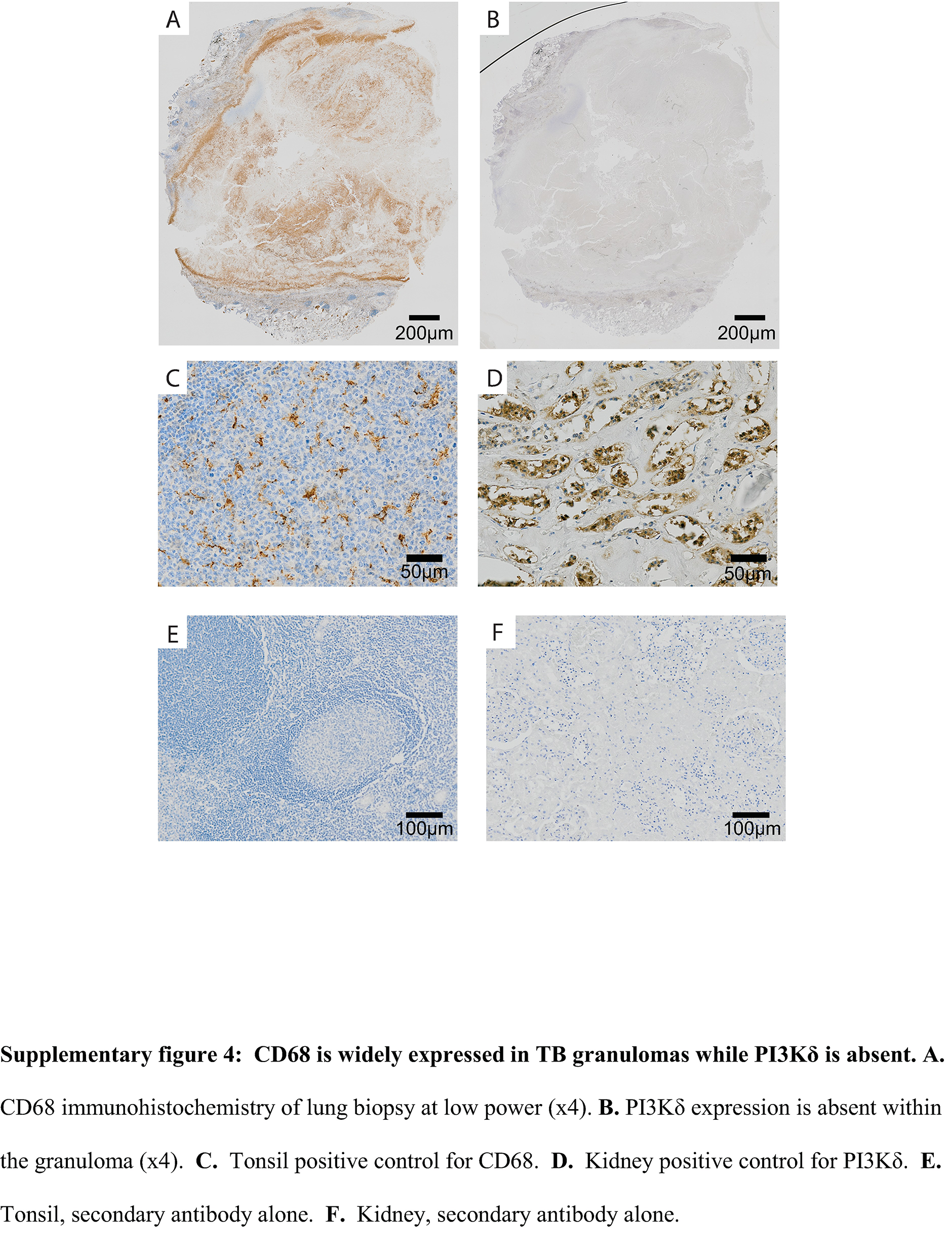

Supplement: S4 Fig — A. CD68 immunohistochemistry of lung biopsy at low power (x4). B. PI3Kδ expression is absent within the granuloma (x4). C. Tonsil positive control for CD68. D. Kidney positive control for PI3Kδ. E. Tonsil, secondary antibody alone. F. Kidney, secondary antibody alone. (TIF) [file ppat.1006367.s004.tif]

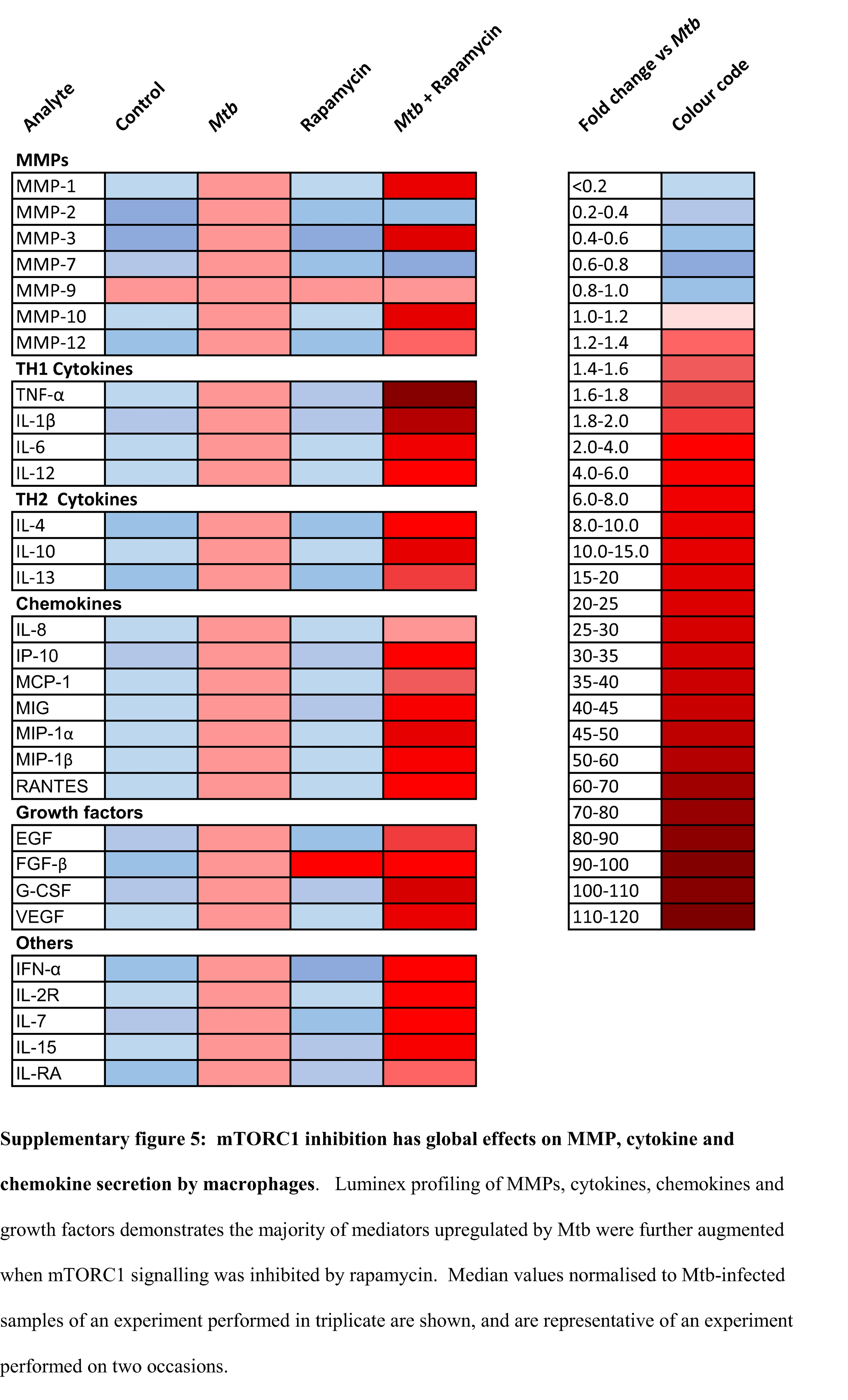

Supplement: S5 Fig — Luminex profiling of MMPs, cytokines, chemokines and growth factors demonstrates the majority of mediators upregulated by Mtb were further augmented when mTORC1 signalling was inhibited by rapamycin. Median values normalised to Mtb-infected samples of an experiment performed in triplicate are shown, and are representative of an experiment performed on two occasions. (TIF) [file ppat.1006367.s005.tif]

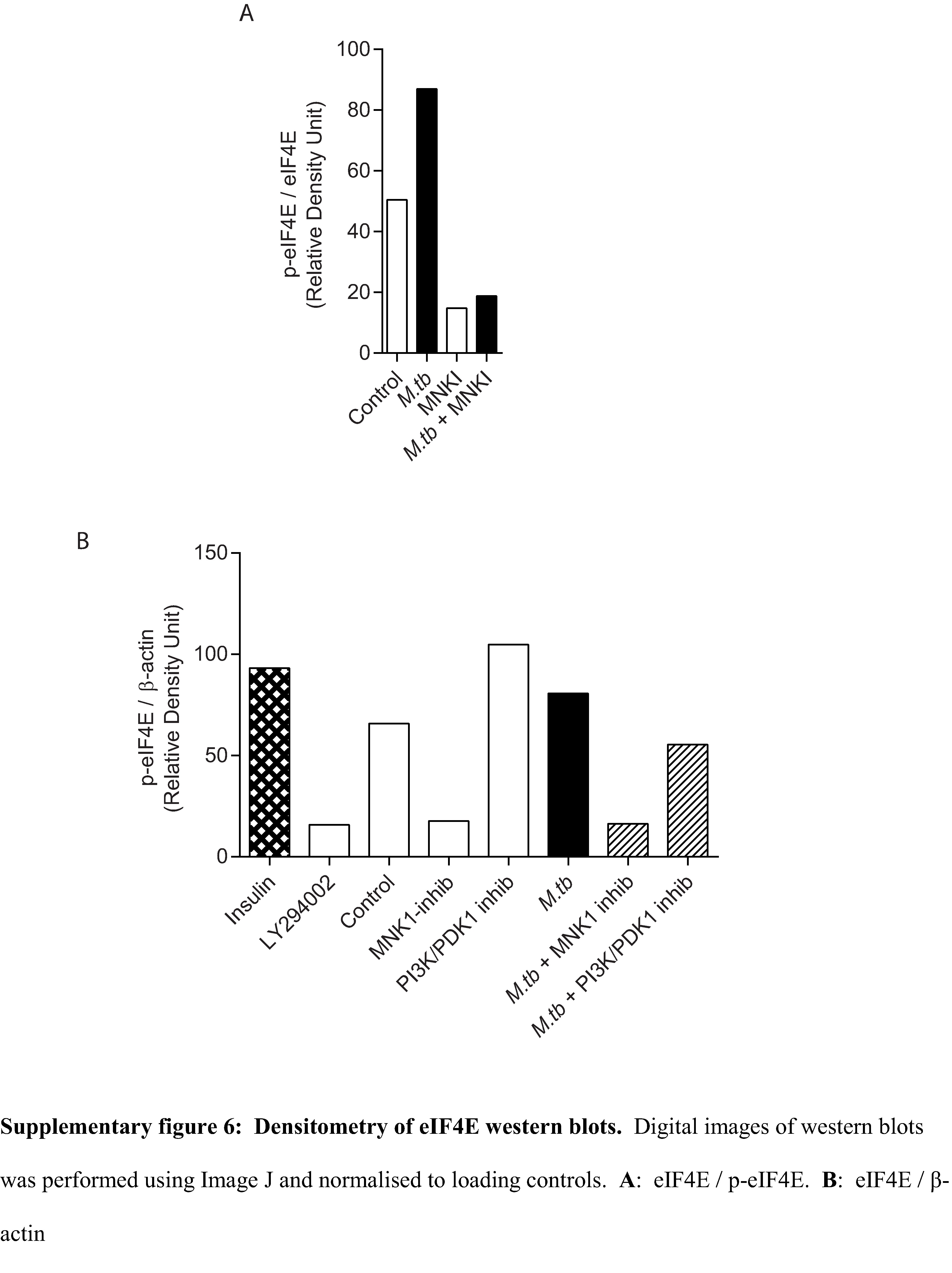

Supplement: S6 Fig — Digital images of western blots was performed using Image J and normalised to loading controls. A: eIF4E / p-eIF4E. B: eIF4E / β-actin (TIF) [file ppat.1006367.s006.tif]

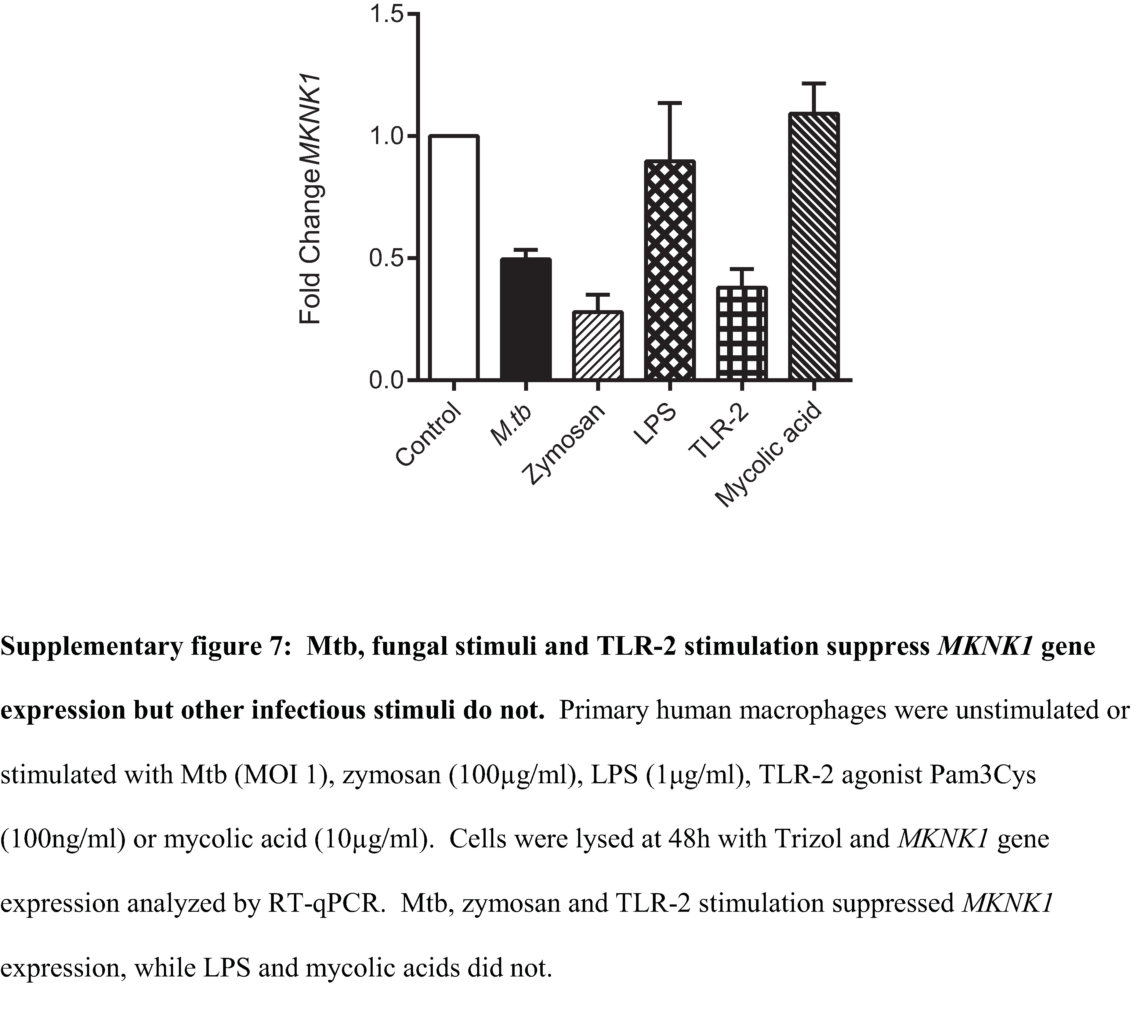

Supplement: S7 Fig — Primary human macrophages were unstimulated or stimulated with Mtb (MOI 1), zymosan (100μg/ml), LPS (1μg/ml), TLR-2 agonist Pam3Cys (100ng/ml) or mycolic acid (10μg/ml). Cells were lysed at 48h with Trizol and MKNK1 gene expression analyzed by RT-qPCR. Mtb, zymosan and TLR-2 stimulation suppressed MKNK1 expression, while LPS and mycolic acids did not. (TIF) [file ppat.1006367.s007.tif]
